# Strongly confined localized surface plasmon resonance (LSPR) bands of Pt, AgPt, AgAuPt nanoparticles

**DOI:** 10.1038/s41598-019-53292-1

**Published:** 2019-11-12

**Authors:** Mao Sui, Sundar Kunwar, Puran Pandey, Jihoon Lee

**Affiliations:** 1 Institute of Hybrid Materials, College of Materials Science and Engineering, Qingdao University, Qingdao, 266071 P. R. China; 2 Department of Electronic Engineering, College of Electronics and Information, Kwangwoon University, Nowon-gu Seoul, 01897 South Korea

**Keywords:** Nanoparticles, Nanoparticles, Photocatalysis

## Abstract

Multi-metallic alloy nanoparticles (NPs) can enable the advanced applications in the energy, biology, electronics, optics and catalysis due to their multi-functionality, wide tunable range and electronic heterogeneity. In this work, various mono-, bi- and tri-metallic nanostructures composed of Ag, Au and Pt are demonstrated on transparent *c*-plane sapphire (0001) substrates and the corresponding morphological and optical characteristics are thoroughly investigated. The resulting Pt and AuPt NPs in this study demonstrate much enhanced LSPR responses as compared to the pure Pt NPs from the previous studies, which was contributed by the synergistic effect of Au and Pt and improved surface morphology. These results are sharply distinct in terms of surface morphology and elemental variability from those obtained by the dewetting of monometallic Ag, Au and Pt films under the similar growth conditions, which is due to the distinct dewetting kinetics of the bi-layer and tri-layer films. These NPs exhibit strongly enhanced localized surface plasmon resonance (LSPR) bands in the UV-VIS wavelengths such as dipolar, quadrupolar, multipolar and higher order resonance modes depending upon the size and elemental composition of NPs. The LSPR bands are much stronger with the high Ag content and gradually attenuated with the Ag sublimation. Furthermore, the VIS region LSPR bands are readily blue shifted along with the reduction of NP size. The Ag/Pt bi-layers and Ag/Au/Pt tri-layers are systematically dewetted and transformed into various AgPt and AgAuPt nanostructures such as networked, elongated and semispherical configurations by means of enhanced surface diffusion, intermixing and energy minimization along with the temperature control. The sublimation of Ag atoms plays a significant role in the structural and elemental composition of NPs such that more isolated and semispherical Pt and AuPt NPs are evolved from the AgPt and AgAuPt NPs respectively.

## Introduction

Nanoparticles (NPs) composed of various metallic elements have been attracting significant attentions in the field of catalysis, sensing, biology, energy and electronics because of their superior characteristics compared to their monometallic counterparts^1–5^. Multi-metallic alloy NPs can provide proficient advantages over the monometallic NPs: i.e. the multi-functionality, site specific response, dynamic surface plasmon resonance and electronic heterogeneity induced by the elemental variation^6–9^. For instance, the ultra-fine PdAg alloy NPs have exhibited the excellent catalytic activity and selectivity as compared to the Pd NPs due to the bimetallic synergy between Pd and Ag such that the Pd offers the hydrogenation activity while the Ag improves the selectivity of the target^10^. Furthermore, apart from the manipulation of size, density and structure, the variation of elemental composition in the alloy NPs can offer an additional parameter to tune the physical and chemical properties of NPs^11–13^.

Nowadays, one of the most extensively exploited features of metallic NPs is the localized surface plasmon resonance (LSPR), which refers to the collective oscillation of electrons on the metallic NPs excited by the incident photons at the resonant frequency^14^. The excitation of LSPR bands enables the enhanced and tunable electromagnetic fields, light absorption and scattering based on the physical and elemental parameters of NPs, which in turn holds the promise of enhanced device performances^15^. Owing to the strong LSPR responses, the Ag and Au NPs have been widely investigated for the plasmonic energy, catalysis, photonics and sensing applications^16–18^. On the other hand, the Pt NPs have been the promising candidates for the catalytic applications due to its strong interaction with the surrounding and chemical stability but lack of the strong LSPR response such as in the Ag and Au NPs^19,20^. By referencing these persuasive advantages of each NP, the fabrication of alloy NPs consisting of Au, Ag and Pt can be of significant benefit for many practical applications. However, the systematic study on the growth characteristic, evolution of morphology and optical responses of the AgPt and AgAuPt alloy NPs by using the solid state dewetting approach has not been demonstrated yet.

Multi-metallic alloy NPs applied in various application are usually fabricated by using the chemical synthesis and nanolithography, which could provide the fine control over the NP size, shape and elemental composition^21,22^. However, the chemical synthesis approach yields the metallic NPs in the form of colloidal solutions, which involves the complex reactants during the synthesis and transformation on the substrates. Thus, the corresponding device performance can be challenged by the residual impurities and stability. Meanwhile, the nanolithography approach could provide the large scale self-assembly of metallic alloy NPs, but it has the limitation of complex process due to the resolution limit of lithography techniques and also requires the highly sophisticated instrumentations. Thus, the fabrication and detailed characterizations of various multi-metallic AgPt, AgAuPt and AgAuPt NPs by the solid-state dewetting approach can be of great importance for many practical applications but has not been demonstrated in detail up to date.

**Figure 1:**
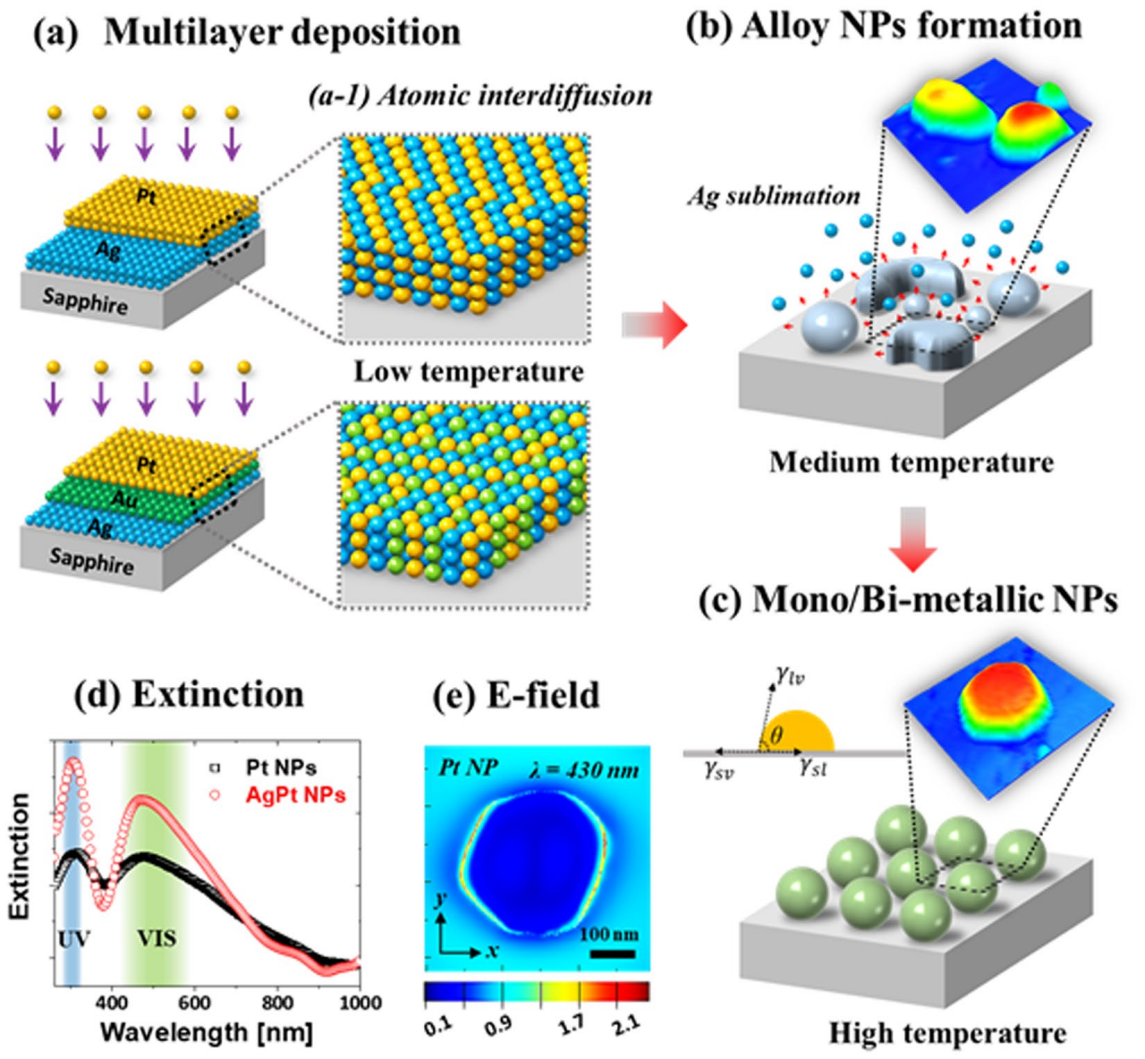
Fabrication process of monometallic and multimetallic alloy NPs along with the concurrent effect of interdiffusion, dewetting and sublimation. (**a**) Schematics of deposition of Ag/Pt and Ag/Au/Pt multilayers on sapphire (0001). (a-1) Atomic interdiffusion at low annealing temperature. (**b**) Formation of alloy nanoparticles (NPs) and concurrent sublimation of Ag atoms. (**c**) Formation of Pt and AuPt NPs after Ag sublimation. (**d**) Comparison of extinction of typical AgPt and Pt NPs. (**e**) Local e-field distribution of typical Pt NP by the finite difference time domain (FDTD) simulation.

In this work, the AgPt and AgAuPt alloy NPs are fabricated by using the solid-state dewetting approach, followed by the physical vapor deposition of various bi- and tri-layers of Ag, Au and Pt atoms on sapphire (0001). The effects of size and elemental variation of NPs on the LSPR properties are also systematically exploited in the UV-VIS-NIR spectra, which shows the tunable and dynamic LSPR responses. The dewetting characteristic, surface morphology evolution and corresponding LSPR properties are significantly distinct from those of monometallic counterparts. By the systematic annealing control of deposited Ag/Pt bi-layers and Ag/Au/Pt tri-layer films as shown in Fig. 1, various shape, size, density and elemental composition of AgPt and AgAuPt alloy nanostructures are realized respectively. Temperature induced surface diffusion and interdiffusion of atoms, nucleation, surface energy minimization and equilibrium crystal configuration govern the growth of alloyed NPs. Furthermore, the sublimation of Ag also plays an important role in the evolution of surface morphology and elemental composition of NPs, which results in the Pt and AuPt NPs at high temperature.

## Results and Discussion

Figure 1 shows the fabrication process of mono- and multi-metallic NPs of Ag, Au and Pt by the annealing of Ag/Pt bi-layer and Ag/Au/Pt tri-layer films. Upon annealing, the metallic films on a substrate can transform into the isolated NPs based on the solid-state dewetting at temperatures below the melting points of elements^23^. Generally, the deposited thin films at an ambient temperature are unstable or metastable due to the insufficient surface diffusion to attain low energy configuration, and the adatoms agglomerate into the isolated particles when annealed to a sufficient temperature^24^. Depending upon the degree of diffusion, interaction with the substrate and growth conditions, various surface morphologies of pure Ag^25^, Au^26^ and Pt^27^ nanostructures have been successfully demonstrated on sapphire (0001). For instance, while the Pt films showed minor surface roughness evolution without the formation of definite nanostructures up to 600 °C, the Ag and Au films formed well-developed semi-spherical NPs even at ~ 400 °C due to much high diffusivity of Ag and Au atoms. In the case of multi-layer films, the dewetting extent and resulting surface nanostructures can be largely distinct due to the difference in the surface diffusivity, interdiffusion and miscibility, surface energies and interfacial energies of metallic atoms^28^.

In this study, the deposition sequence was chosen according to the diffusivity of materials: i.e. the Ag layer first and then the Au and Pt in the case of the Ag/Au/Pt bi-layers as shown in Fig. 1(a). With the Ag/Pt and Ag/Au/Pt deposition configuration, the overall dewetting process can be enhanced owing to the high surface diffusivity and low surface energy of Ag atoms in the underlayer. The surface energy of Ag, Au and Pt are 1250, 1500 and 2475 mJ m^− 2^ respectively^29^. When the Pt layer was deposited on the Ag layer, there forms the Ag/Pt interface and upon annealing, some of the Ag and Pt atoms may enter into the existing vacancies at the interface, giving a rise to the partially intermixed interface. By further annealing, the atomic intermixing can be more enhanced and thus can result in the formation of fully alloyed bimetallic layer with the increased global diffusivity of atoms as depicted in Fig. 1(a-1) ^30^. In the case of Ag/Au/Pt configuration, the interdiffusion of atoms can simultaneously occur at the both Ag/Au and Au/Pt interfaces. Due to the increased disparity in the diffusivity between atoms, the intermixing process can be further complicated at the initial stage of annealing. Consequently, at a sufficiently high temperature, the dewetting can be initiated by the nucleation of pinholes at the low energy sites. The structural and elemental transformation of the AgPt and AgAuPt alloy NPs at high temperature can be simultaneously affected by the degree of dewetting as well as the Ag atom sublimation as shown in Fig. 1(b). Due to the higher vapor pressure of Ag atoms, the Ag atoms preferentially can desorb from the NP matrix and the rate of Ag sublimation exponentially increases with the temperature. As a result, annealing at above a critical temperature may lead to the formation of pure Pt NPs or AuPt NPs as shown in Fig. 1(c) due to the extensive sublimation of Ag atoms^31,32^. The final structure of Pt and AuPt NPs can be significantly varied due to the difference in the atomic diffusivity, surface energy and equilibrium configuration. The morphological and elemental variation of surface NPs were directly reflected in the optical properties as shown in Fig. 1(d),(e), which clearly demonstrated the distinctive LSPR intensity, peak position and local e-field distribution. The detailed morphological, elemental and optical analysis of various mono- and multi-metallic alloy NPs have been systematically studied in the following sections.

**Figure 2:**
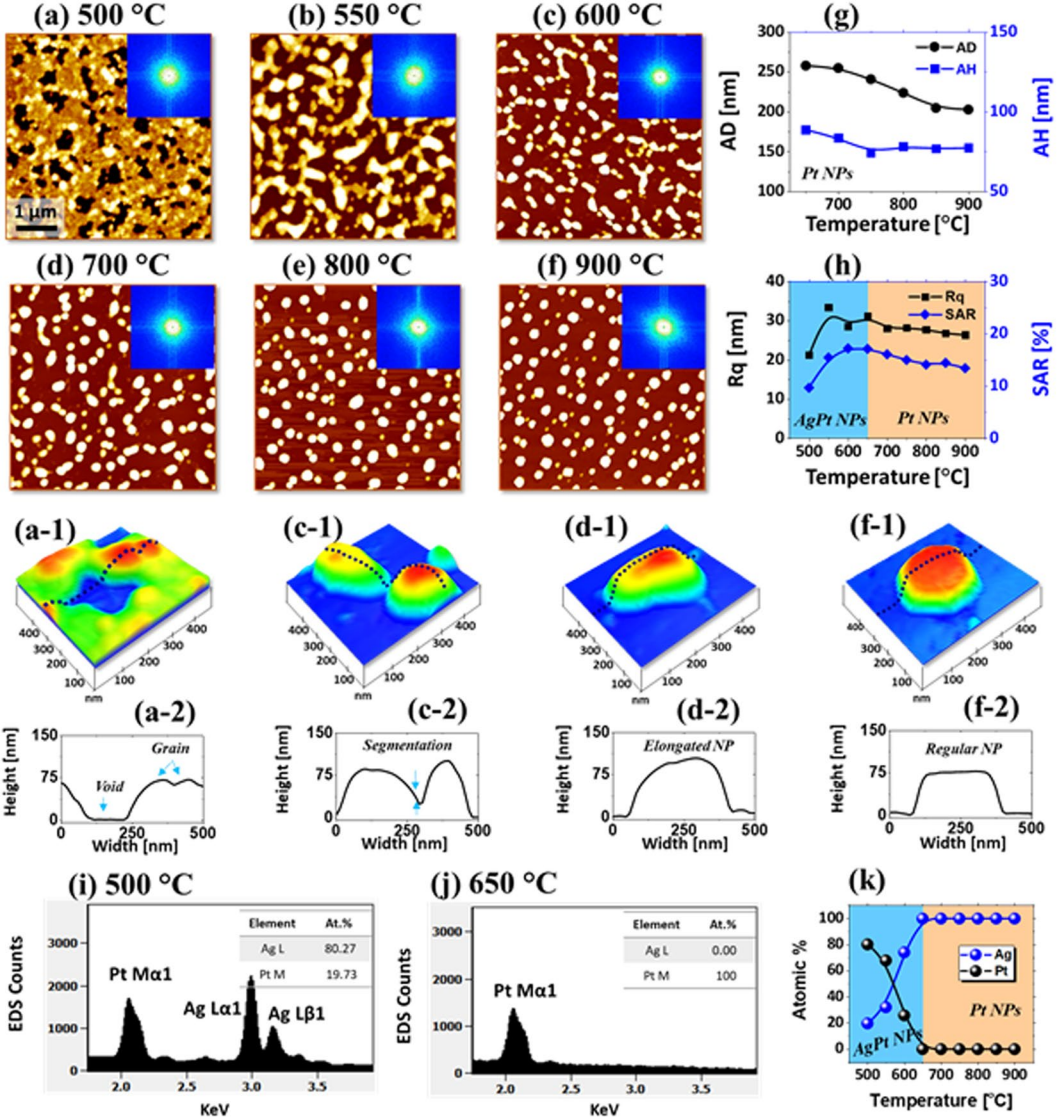
Evolution of AgPt nanoclusters and Pt NPs by the dewetting of Ag_20nm_/Pt_10nm_ bi-layer films on sapphire (0001) based on annealing between 500 and 900 °C for 120 s. (**a**–**f**) AFM top-views (5 × 5 µm^2^) with Fourier filter transform (FFT) power spectra as insets. (a-1) – (f-1) Enlarged AFM 3D side-views of typical NPs at different temperature. (a-2) – (f-2) Corresponding line profiles across the typical NPs. (**g**) Summary plots of average diameter (AD) and average height (AH). (**h**) Summary plots of RMS roughness (Rq) and surface area ratio (SAR). (**i,j**) Energy dispersive x-ray spectroscope (EDS) spectra of the AgPt and Pt NPs. (**k**) Summary plots of atomic (at) % of Ag and Pt.

Figure 2 presents the detailed morphological and elemental evolution of the Ag_20nm_/Pt_10nm_ bi-layers annealed between 500 and 900 °C for 120 s. In general, the growth of voids, three-dimensional nanoclusters and semi-spherical NPs were gradually observed along with the annealing. The dewetting process was already initiated at 500 °C with the formation of large voids of ~ 200 to 500 nm and granular features as shown by the AFM top-view in Fig. 2(a). The dewetting degree was significantly enhanced as compared with the pure Pt film while it was reduced from the pure Ag film of similar thickness^25,27^. The typical AgPt nanoclusters are shown by the AFM side-view and line profile in Fig. 2(a-1),(a-2). With the increment in temperature, the voids were grown larger due to the coalescence with the adjacent and the diffusing atoms started to agglomerate into the 3D nanoclusters. This resulted in the evolution of interconnected AgPt nanoclusters of ~ 75 nm average (AH) and ~ 300 nm average diameter (AD) at 550 °C as observed in Fig. 2(b). The void growth can be attributed to the coalescence growth in order to minimize the interfacial energy between the film and substrate^31^. When the temperature was increased above 600 °C, a sharp transition in the surface morphology was observed as the fragmentation of networked nanoclusters occurred and mostly isolated NPs were formed due to the Rayleigh-like instability^33^. From the cross-sectional line profiles in Fig. 2(c-2), the average height (AH) and diameter (AD) of the isolated irregular AgPt NPs were found to be ~ 80 and 270 nm respectively. Both RMS roughness (Rq) and surface area ratio (SAR) were generally increased with the evolution of isolated nanoclusters up to 600 °C as shown in Fig. 2(h).

During the dewetting process, the sublimation of Ag atoms can play a major role in the evolution of surface nanostructures, which was confirmed by the elemental analysis as shown in Fig. 2(i)–(k). The full range EDS spectra of each sample are provided in Fig. S4. The EDS peaks of Al, O and Pt were commonly detected for all samples while the Ag peaks were only detected up to 600 °C. Specifically, the Ag Lα1 and Ag Lβ1 peaks were significantly attenuated between 500 and 650 °C, suggesting the accelerated sublimation of Ag atoms from the alloy nanostructures as clearly shown in Fig. 2(i)–(k) ^31,32^. In addition, the atomic % of Ag and Pt were 80.27 and 19.73% at 500 °C, which was changed to 0 and 100% at 650 °C. This clearly indicates that the Ag atoms were completely desorbed from the NP matrix and pure Pt NPs were resulted above 650 °C. When the temperature was increased above 600 °C, the growth of NPs was mainly contributed by the shape transformation. For instance, the irregular-isolated NPs were gradually transformed into the semi-spherical and then hexagonal shape as shown in Fig. 2(d)–(f), which can be described by the reduction of surface energy in order to gain the equilibrium configuration of nanocrystals^34^. The final surface morphology of NPs can be determined by the surface and interface energy and the thermodynamic equilibrium. The AH was slightly decreased and remained constant ~ 75 nm whereas the AD was decreased to ~ 225 nm between 650 and 900 °C as shown in Fig. 2(g). Similarly, the Rq and SAR were gradually decreased as the size of NPs was decreased along with the annealing. These isolated pure Pt NPs above 650 °C demonstrated much improved size, spacing and configuration from those obtained with the pure Pt film dewetting due to the enhanced diffusion by the Ag atoms^27^.

**Figure 3:**
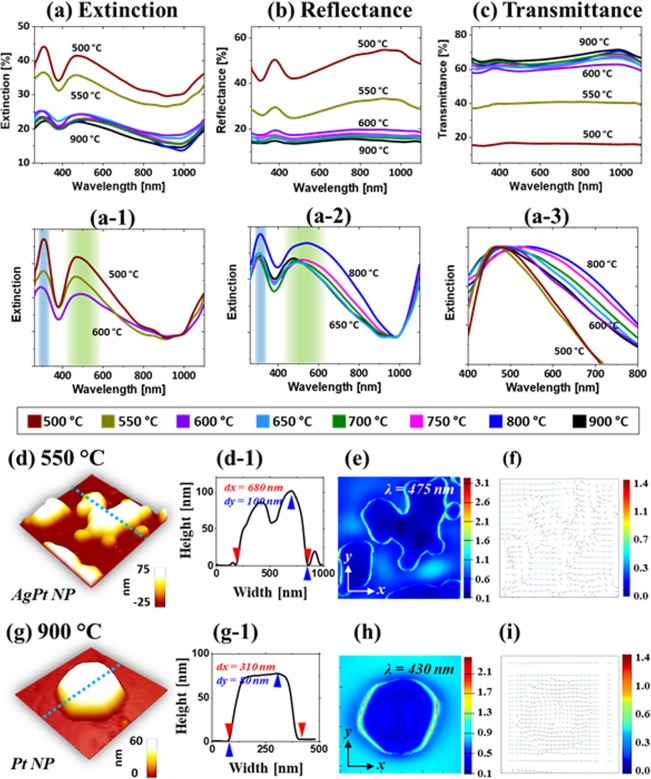
Optical characterization of the AgPt and Pt NPs fabricated with the Ag_20nm_/Pt_10nm_ bi-layers at different temperatures. (**a**) – (a-3) Extinction and normalized extinction spectra. (**b**) Reflectance spectra. (**c**) Transmittance spectra. The reflectance and transmittance spectra in (**a,b**) are experimentally measured and the extinction spectra in (**c**) are extracted: E % = 100% - (R + T) %. (**d**) and (**g**) AFM side-view of the AgPt and Pt NPs selected for finite difference time domain (FDTD) simulations. (d-1) and (g-1) Cross-sectional line profiles across the NP in (**d**) and (**g**). (**e**) and (**h**) e-field distribution in irregular AgPt NP and hexagonal Pt NP. (**f**) and (**i**) Corresponding e-field vector plots.

Figure 3 shows the optical properties of AgPt and Pt NPs fabricated with the Ag_20nm_/Pt_10nm_ bi-layers. The structural and elemental variation of nanostructures give rise to the diverse LSPR properties as seen in the extinction, reflectance and transmittance spectra between 300 and 1100 nm in Fig. 3(a)–(c). In addition, the measured optical spectra were normalized and the FDTD simulation was employed to observe the local e-field distribution. As shown in Fig. 3(a), in general, the extinction spectra clearly demonstrated the distinct spectral shape at below and above 600 °C. In specific, the large AgPt nanoclusters (<600 °C) exhibited the strong extinction peaks in the UV and VIS regions whereas the spherical Pt NPs (>650 °C) showed the relatively weaker and broader UV and VIS peaks as seen in Fig. 3(a-1)(a-2). The extinction peaks in the UV and VIS can be assigned to the excitation of multi-polar resonance (MR) in the VIS and higher order resonance (HR) in the UV region^35,36^. Because of the large size of AgPt and Pt NPs, the peaks in the VIS can be mostly contributed by the MR. The LSPR peaks became weaker and broader as shown in Fig. 3(a-1) as the plasmonic resonance can be hindered by the sublimation of Ag atoms. The local e-field distribution for the typical AgPt NP on sapphire is shown in Fig. 3(d)–(f) by the FDTD simulation, which clearly demonstrates the strong e-field confinement on the surface at the resnance wavelength. It was also observed that the e-field vectors were directed in the multiple directions along the surface of NPs in Fig. 3(f), likely due to the excitation of multiple resonance bands. In addition, the simulated extinction power spectrum for the typical AgPt nanocluster is presented in Fig. S5(c). In fact, the measured extinction spectra exhibited the stronger peaks in the UV and VIS region while the simulated extinction showed a flatter response, which could due to the wide disparity in the size distribution of NPs.

With the formation of isolated Pt NPs (>650 °C), the extinction spectra showed much wider and weaker peaks in the VIS region (450–600 nm) and UV region (~320 nm) as shown in Fig. 3(a-2). The broadening effect of the VIS peaks along with the temperature was traced in Fig. 3(a-3), which can be correlated to the Ag sublimation as discussed. Furthermore, the corresponding e-field profile, vector plot and extinction spectrum of the typical Pt NP are shown in Fig. 3(g)–(i) and Fig. S5. Although the e-field was strongly concentrated at the NPs/sapphire interface, the overall e-field intensity was clearly decreased from the AgPt alloy NPs. Since the size of Pt NP was significantly reduced form the AgPt alloy NPs, the VIS extinction peak can be mainly contributed by the dipolar resonance although other higher order resonance modes can still be present. The e-field vector plot clearly shows that the e-field vector are mostly along the axis of NPs while few were still directed in multiple directions.

From the reflectance spectra of AgPt and Pt NPs in Fig. 3(b), two dips in the UV region (~450 nm) and VIS region (~500 nm) were observed corresponding to the resonance modes of the AgPt nanoclusters and Pt NPs. The absorption dips of the AgPt NPs were gradually attenuated and became flatter with the sublimation of Ag atoms as discussed^36,37^. In the case of transmittance spectra in Fig. 3(c), they also showed distinct behaviors for the large AgPt NPs and isolated semi-spherical Pt NPs. The large AgPt NPs exhibited almost flat transmittance spectra, which can be due to the pronounced forward scattering with the relatively strong MR mode of the large AgPt nanoclusters as discussed^38^. And with the evolution of isolated and semispherical Pt NPs, the absorption dips in the VIS region were appeared, which can be correlated to the strong dipolar resonance with the reduced size of Pt NPs^35,36^. As NPs became smaller, the strong influence of the quad-polar resonance in the MR mode can be gradually reduced and the effect of dipolar resonance can begin to appear such as in the reduced forward scattering, resulting in the formation of absorption dips. In comparison to the pure Pt NPs from the previous study^27^, the LSPR properties in this study were significantly enhanced in the visible wavelength, which can be correlated to the improved configuration and uniformity of Pt NPs.

**Figure 4:**
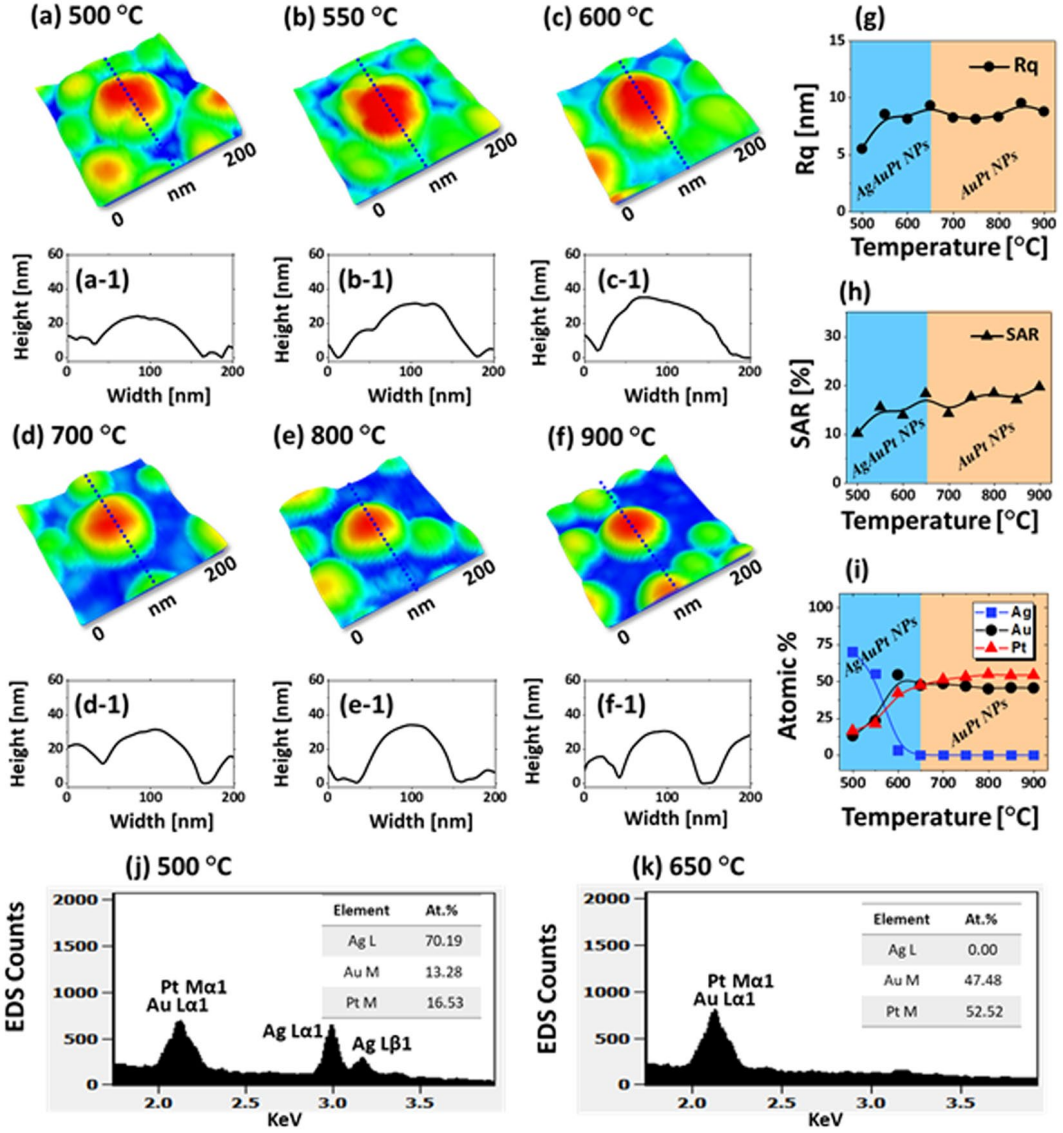
Fabrication of small size AgAuPt and AuPt NPs with the Ag_8.25nm_/Au_2.25nm_/Pt_2.25nm_ tri-layers by annealing between 500 and 900 °C for 120 s. (**a–f**) AMF side-views (200 × 200 nm^2^). (a-1) – (f-1) Cross-sectional line profiles. (**g–i**) Summary plots of Rq, SAR and at % of Ag and Pt at specific temperatures. (**j,k**) EDS spectra of the AgAuPt and AuPt NPs at 500 and 700 °C respectively. Insets show the composition of Ag and Pt in at %.

Figure 4 presents the fabrication of small AgAuPt and AuPt alloy NPs with the Ag_8.25nm_/Au_2.25nm_/Pt_2.25nm_ tri-layers annealed between 500 and 900 °C for 120 s. In this set, thinner Ag, Au and Pt layers were deposited in a sequence, which constructed the Ag/Au and Au/Pt interfaces. As discussed, the Ag, Au and Pt atoms can be intermixed well at increased temperature and give rise to the alloyed nanoclusters as all these atoms do not tend to segregate. In fact, the overall film thickness was reduced in this case, which can facilitate the dewetting process and the void nucleation and growth as well as cluster breakdown can be more favorable with the reduced thickness^39^. The rapid dewetting of thin tri-layers can be discussed by the enhanced surface diffusion and intermixing of Ag, Au and Pt atoms. This results in the formation of more isolated and regular alloy NPs even at lower temperature. In general, much smaller and isolated NPs were obtained between 500 and 900 °C as presented by the AFM side-views in Fig. 4(a)–(f). The large-scale AFM images and line profiles are provided in Figs S6–S8. In specific, at 500 °C, the irregular AgAuPt alloy NPs of AH ~ 30 nm and AD ~ 120 nm were observed as shown in Fig. 4(a). Between 550 and 600 °C as shown in Fig. 4(b),(c), the AgAuPt NPs were gradually increased in size and spacing due to the enhanced diffusion with temperature. However, the configuration was found to be slightly elongated, which can be correlated to the coalescence growth in the temperature range^34^. The Rq and SAR values were also increased along with the NP size increase as shown in Fig. 4(g),(h). Like the previous set, the growth of alloy NPs was significantly affected by the Ag sublimation. As shown in Fig. 4(i)–(k), the Ag atomic % was sharply reduced between 500 and 600 °C and then became zero at above temperature. Due to the Ag atom sublimation, the transformation in the surface morphology can be more dramatic. As the temperature was increased between 700 and 900 °C, the areal density of NPs was increased and the shape became much spherical as shown in Figs 4(d)–(f) and S4–S4. The density increment of NPs can be correlated to the fragmentation of large NPs due to the Rayleigh-like instability^33^. On the other hand, the spherical shape of NPs can be attributed to the tendency to minimize the surface energy and gain equilibrium configuration as discussed. The isolated NPs generally had the similar AH height of ~ 35 nm whereas the AD was gradually decreased from 100 to 60 nm as seen in the line profiles of typical NPs in Fig. 4(d-1)–(f-1). The Rq of NPs at high temperature showed similar value as the average height was similar while the SAR was mildly increased due to the higher density of NPs. From the EDS spectra in Fig. 4(k), the Ag peaks were not detected above 650 °C, indicating the complete sublimation of Ag atoms and formation of AuPt alloy NPs. Thus, the size reduction of NPs at high temperatures can also be correlated to the Ag sublimation.

**Figure 5:**
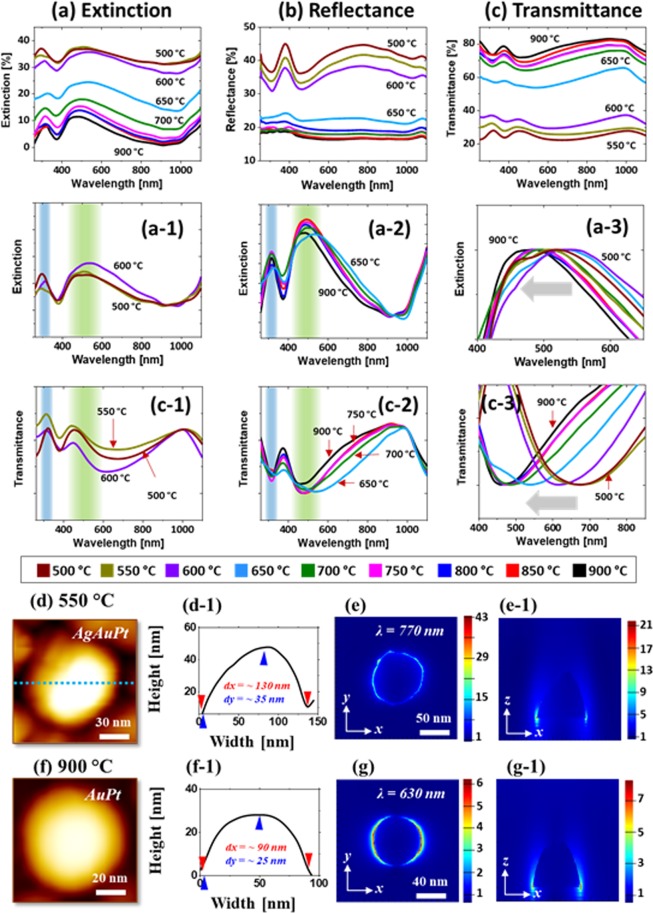
Optical analysis of the small AgAuPt and AuPt NPs fabricated with the Ag_8.25nm_/Au_2.25nm_/Pt_2.25nm_ tri-layers. (**a**) – (a-3) Extinction and normalized extinction spectra. (**b**) Reflectance spectra. (**c**) – (c-3) Transmittance and normalized transmittance spectra. (**d**) and (**e**) AFM images of typical AgAuPt and AuPt NPs chosen for FDTD simulation. (d-1) and (f-1) Cross-sectional line profiles across the NP in (**d**) and (**f**). (**e**) and (**g**) E-field distribution in xy-plane. (e-1) and (g-1) E-field distribution in xz-plane.

Figure 5 shows the corresponding LSPR properties of small and dense AgAuPt and AuPt alloy NPs fabricated with the Ag_8.25nm_/Au_2.25nm_/Pt_2.25nm_ tri-layers. As compared to the AgPt and Pt NPs in the previous set, this set exhibited the distinct LSPR properties. Furthermore, within a set, the optical spectra varied largely along with the evolution of surface morphology and elemental composition of alloy NPs. From the morphological and elemental analysis, the NPs were generally denser and less than 200 nm in the AD and 40 nm in the AH and consisted of AgAuPt up to 600 °C and AuPt at above temperature. From the extinction spectra in Fig. 5(a), it again generally showed a grouped behavior with the AgAuPt and AuPt NPs. The peaks in the VIS and UV regions can be correlated to the dipolar resonance (DR) and quad-polar resonance (QR) of the smaller NPs^33,40^. The AgAuPt NPs showed comparatively weaker extinction peaks compared with the large AgPt nanoclusters in the previous set likely due to the less Ag content and smaller size^36,37^. Despite the sublimation of Ag at 600 °C, extinction peaks were still observed, which can be likely due to Au component. The local e-field profiles of typical alloy NP is presented in Fig. 5(d),(e), which showed a strong e-field confinement at the boundary of NPs with the much smaller size. From the simulation of extinction power spectra, a strong DR mode was observed at ~ 770 nm and a minor QR shoulder at ~ 400 nm as shown in Fig. S9. The simulated extinction spectrum for the typical AgAuPt NP showed a large red shift in the DR peak position as compared to the experimental result. This can be again due to the much wider size distribution of NPs in the real sample and in addition, the AFM scanned NPs size could be larger than the real size due to the tip effect.

When the smaller AuPt NPs were formed after the sublimation of Ag atoms, the DR peaks was readily blue shifted to ~ 485 nm as presented in Fig. 5(a-2). However, the QR peaks didn’t show much noticeable changes with the changes in size and elemental composition of the NPs. At the same time, since the uniformity of AuPt alloy NPs was improved with temperature, the LSPR peaks gradually became narrower. The DR peaks of the small and dense AuPt NPs were significantly narrower from that of the widely spaced larger Pt NPs in the previous set, which can be correlated to the improved uniformity of AuPt alloy NPs as well as the contribution of Au content. The overall shift of DR peak is presented in Fig. 5(a-3), which showed the gradual blue shift of peak position from ~ 520 nm to 470 nm with the reduction of NPs size. From the e-field distribution of AuPt NPs at 900 °C in Fig. 5(f)–(g), it also showed a strong e-field confinement at the boundary of NPs due to the DR resonance mode. The e-field intensity was sharply reduced as compared to the AgAuPt and the resonance peak was blue shifted due to the smaller size along with the removal of Ag component.

In addition, the reflectance spectra in Fig. 5(b) also exhibited a distinct behavior as compared to the previous set. Specifically, the AgAuPt alloy NPs below 600 °C showed a dip in the UV and VIS regions followed by the strong shoulder in the VIS-NIR. These reflectance dips can be correlated to the absorption enhancement in the UV and VIS regions by the DR and QR modes respectively, whereas the shoulder can be developed due to the enhanced backscattering effect of small NPs^38^. In contrast to the large AgPt alloy NPs, these dips were slightly blue shifted due to the smaller size of NPs. With the sublimation of Ag and formation of smaller AuPt NPs above 650 °C, the UV and VIS dips were disappeared. Based on the stronger DR of smaller AuPt alloy NPs, the enhanced backscattering can occur, which may result in the attenuation of absorption dips^38^. The transmittance spectra in Fig. 5(c)–(c-3) also clearly exhibited absorption dips in the UV and VIS regions due to the QR and DR of small and uniform AgAuPt alloy NPs as discussed. Furthermore, the absorption dip intensity was gradually reduced with the sublimation of Ag atoms from the alloy NPs^36^. With much smaller AuPt alloy NPs, the transmittance dips were apparently blue shifted from ~ 670 to 450 nm as shown in Fig. 5(c-3). At the same time, the absorption dips were narrower at high temperature as the uniformity of AuPt alloy NPs were improved.

**Figure 6:**
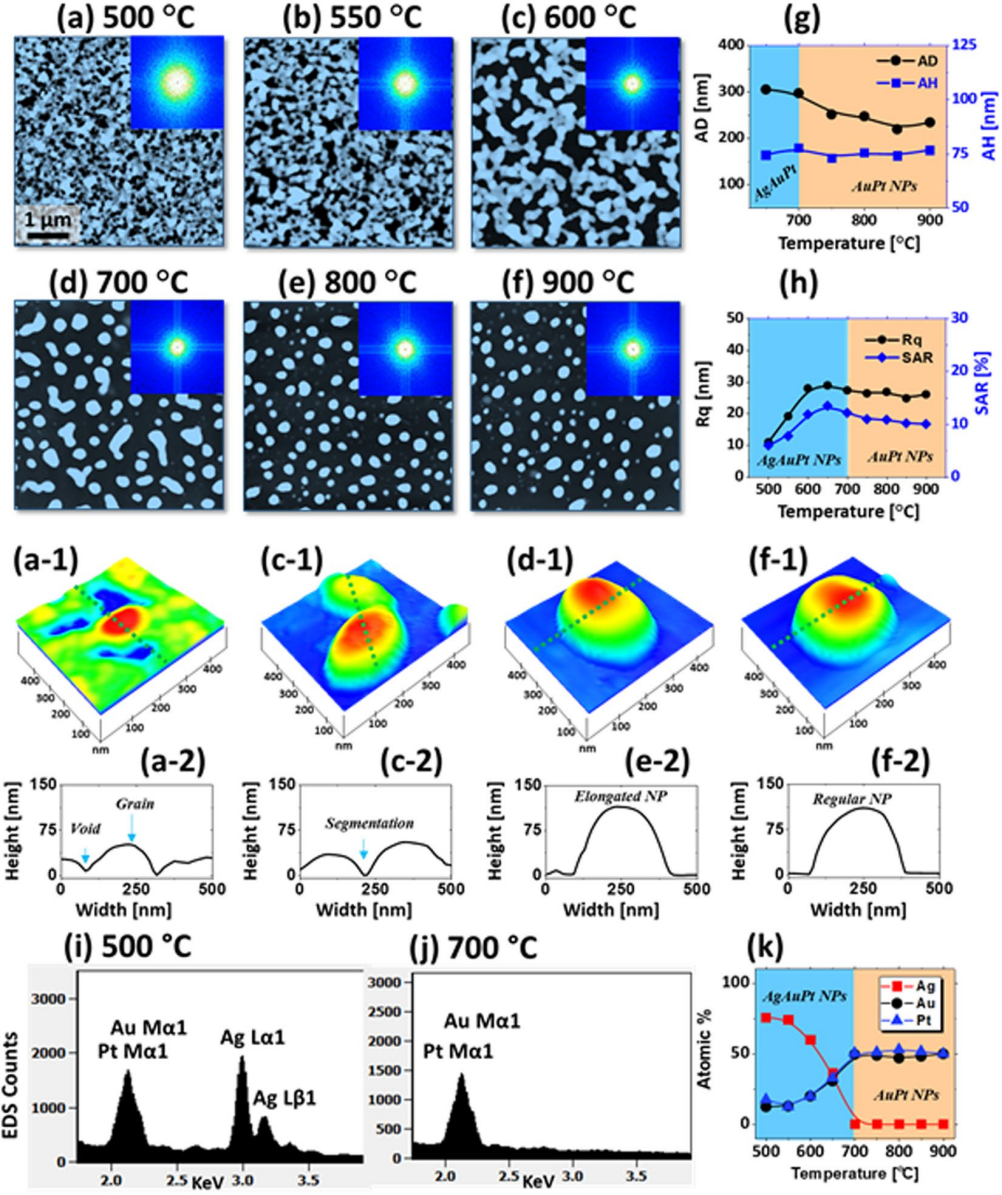
Evolution of large AgAuPt and AuPt NPs on sapphire (0001) with the Ag_16.5nm_/Au_4.5nm_/Pt_4.5nm_ tri-layers annealed between 500 and 900 °C for 120 s. (**a–f**) AFM top-views (5 × 5 µm^2^). Insets show the corresponding FFT patterns. (a-1) – (f-1) Enlarged AFM 3D side-views of typical NPs at different temperature. (a-2) – (f-2) Corresponding line profiles across the typical NPs. (**g**) Summary of AD and AH of isolated AuAgPt and AuPt NPs. (**h**) Summary of Rq and SAR. (**i,j**) EDS spectra of the samples at 500 and 700 °C. (**j**) Summary plots of at % of Ag, Au and Pt.

**Figure 7:**
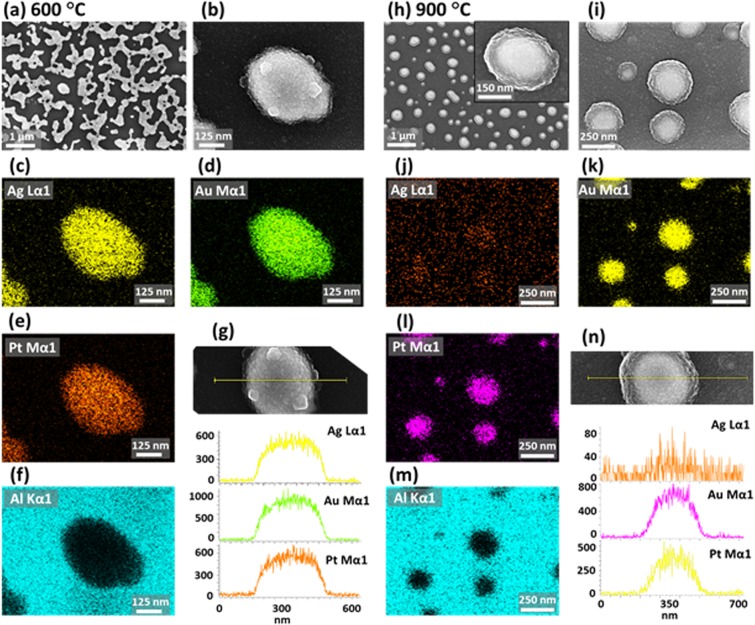
Elemental analysis of the AgAuPt and AuPt NPs fabricated with the Ag_16.5nm_/Au_4.5nm_/Pt_4.5nm_ tri-layers at 600 and 900 °C. (**a,b**) SEM images of AgAuPt NPs. (**c–f**) Elemental maps of Ag, Au, Pt and Al. (**g**) Elemental line profiles through the AgAuPt NP. (**h,i**) SEM images of the AuPt NPs. (**j–m**) EDS maps of Ag, Au, Pt and Al component. (**n**) EDS line profiles on the AuPt NP.

Figure 6 shows the fabrication of large AgAuPt and AuPt NPs with the Ag_16.5nm_/Au_4.5nm_/Pt_4.5nm_ tri-layers by annealing between 500 and 900 °C for 120 s. In this set, the thickness of each metal layer was increased by twice from the previous set in order to investigate the tri-layer film thickness effect on the evolution of NPs. Under the identical annealing condition, the dewetting of thicker tri-layer was significantly altered, which resulted in the formation of larger and isolated NPs. As displayed by the AFM images, although the overall dewetting characteristic was similar with the Ag/Pt (30 nm) bi-layers in the first set, the dewetting degree at specific temperature and surface configurations were largely altered. In fact, the overall thickness of Ag/Au/Pt (25.5 nm) tri-layer films was thinner than the Ag/Pt bi-layer, but the dewetting process was much slower in this set. This can be attributed to the increased number of interdiffusion interfaces and elemental heterogeneity in the multilayer films. Furthermore, the Ag sublimation rate with the AgAuPt alloy system can be much lower because of the high miscibility of Ag and Au as compared to that of Ag and Pt^41,42^. Consequently, the sublimation rate of Ag atoms can be lower with the AgAuPt system, which ultimately can result in the slower growth of voids at low temperature. The surface morphology of AgAuPt and AuPt NPs at different temperatures can be observed in Fig. 6(a)–(f). At 500 °C, the small voids of ~ 50 to 200 nm width and grains of ~ 50 nm AH and ~ 150 nm AD were developed on the film surface. With the increment of temperature, the voids were grown larger by the coalescence with the adjacent voids and the adatoms were agglomerated more compactly. This resulted in the evolution of interconnected AgAuPt nanocluster of ~ 500 nm AD up to 600 °C as shown in Fig. 6(c). By comparing with the first set, the void size was significantly smaller at the initial stage of the dewetting as the growth was hindered, which can be correlated to the slow intermixing and diffusion of atoms in the AgAuPt trimetallic system as discussed. In terms of the Rq and SAR, they were increased with the formation of voids and irregular AgPt nanoclusters between 500 and 600 °C as shown in Fig. 6(h). To investigate the elemental distribution of Ag, Au and Pt, the high-resolution EDS analysis was performed as shown in Fig. 7(a)–(g). Figure 7(a),(b) show the large-scale and enlarged SEM images of AgAuPt NP at 600 °C. The elemental maps and SEM image clearly showed a good match and revealed the homogeneous distribution of Ag, Au and Pt atoms in Fig. 7(b)–(e). Furthermore, the EDS line profile through the NPs as displayed in Fig. 7(f) clearly exhibited the uniform distribution of all three elements in the NP.

At high temperature regime i.e. between 700 and 900 °C, the Rq, SAR, AH and AD also showed decreasing trend as shown in Fig. 6(g),(h). In specific, in this temperature range the AD was reduced from 300 to 225 nm whereas the AH remained almost similar at ~ 75 nm. Meanwhile, there was a slight decrease in areal density as the smaller NPs can be absorbed to the larger ones based on the coalescence growth in order to gain the thermodynamic stability^40^. The evolution of AgAuPt alloy nanoclusters was simultaneously contributed by the self-assembly of the diffusing atoms and Ag sublimation at increasing temperature. As shown in the EDS spectra and at % in Fig. 6(i)–(k), the Ag peaks were completely disappeared above 700 °C and Ag at % became zero, indicating the sublimation of Ag atoms from the AgAuPt alloy nanoclusters. However, the rate of Ag sublimation was lower as compared to the first set and complete sublimation occurred at a higher temperature of 700 °C due to the higher miscibility of Ag and Au as discussed^41,42^. Thus, the elemental composition and size of alloy nanoclusters were significantly altered in this temperature range. The fragmentation of nanocluster occurred at 650 °C and then the isolated NPs gradually became semi-spherical up to 900 °C as shown in Fig. 6(d)–(f), which can be driven by the surface energy minimization and formation of equilibrium configuration with a favorable diffusion of atoms. To confirm the sublimation of Ag and evolution of AuPt NPs, the sample annealed at 900 °C was further analyzed with the EDS mapping as shown in Fig. 7(h)–(n). As observed in the EDS maps and line profiles in Fig. 7(i)–(l), the NPs were formed with the uniform distribution of Au and Pt while the Ag component was not observed. This again indicates the complete sublimation of Ag and formation of the AuPt NPs. By comparing with the previous set, the average density of isolated NPs was significantly reduced whereas the average size was increased by nearly twice due to the increased film thickness.

**Figure 8:**
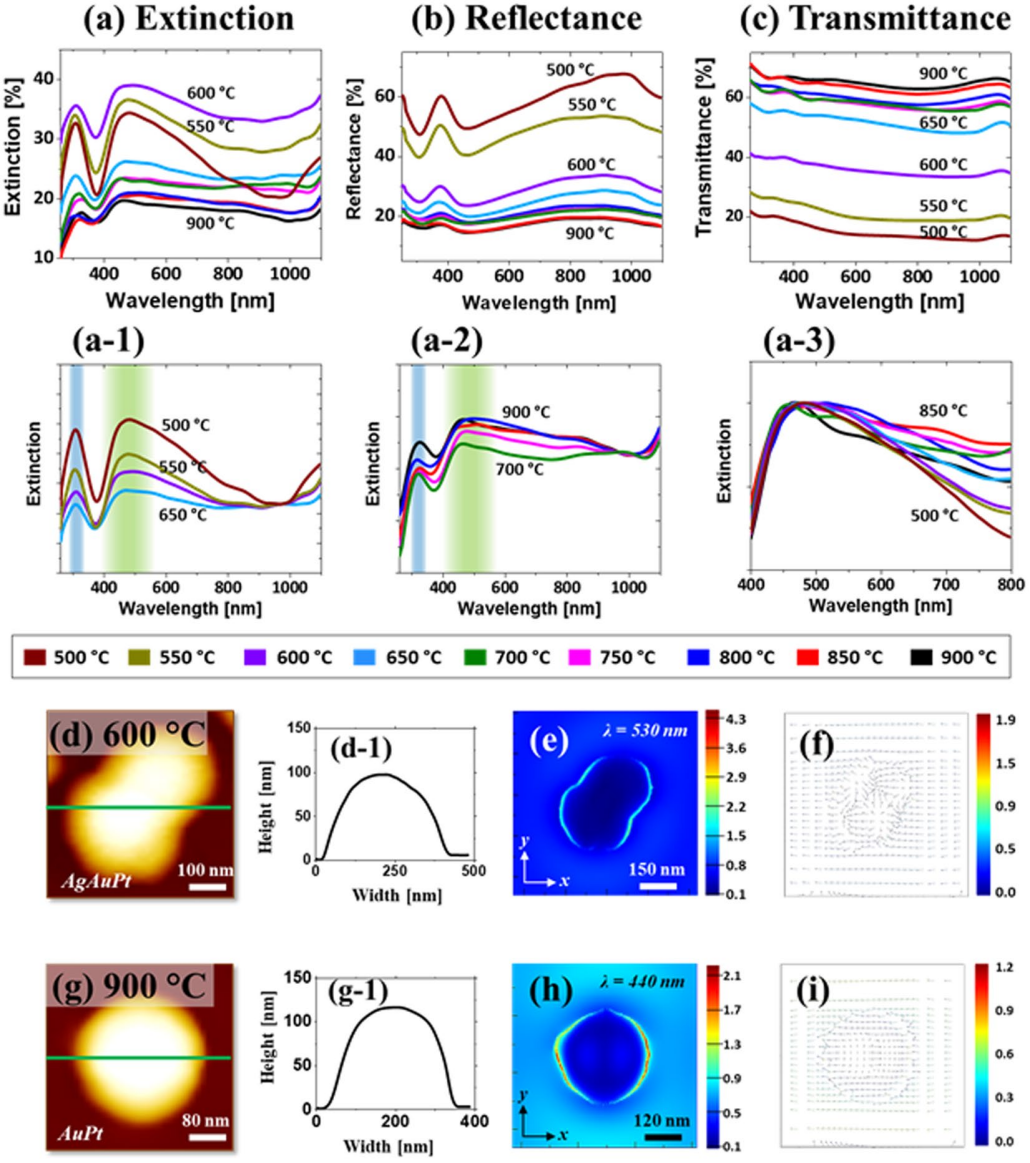
Optical properties of large AgAuPt and AuPt NPs fabricated with the Ag_16.5nm_/Au_4.5nm_/Pt_4.5nm_ tri-layers films. (**a**) – (a-3) Extinction and normalized extinction spectra. (**b**) Reflectance spectra. (**c**) Transmittance spectra. (**d,g**) AFM images of the AuAgPt and AuPt NPs. (d-1) and (g-1) Line profiles of NPs in (**d**) and (**g**). (**e**) and (**h**) E-field profile top-views (x-y plane) at different wavelengths as indicated. (**f**) and (**i**) E-field vector plots.

Figure 8 shows the LSPR properties of large AgAuPt nanoclusters and AuPt NPs fabricated with the Ag_16.5nm_/Au_4.5nm_/Pt_4.5nm_ tri-layers. Although the average size and configurations of these NPs were comparable with the first set, the LSPR properties distinctly varied due to the addition of Au component. Similar to the previous sets, two distinct optical responses were observed for the AgAuPt NPs below 700 °C and AuPt NPs at above. In specific, the extinction spectra in Fig. 8(a),(a-1) demonstrated two strong peaks in the UV and VIS regions with the large and wide coverage AgAuPt nanoclusters. The peak formation in the UV and VIS wavelength of the extinction spectra can be assigned to the resonance of various plasmonic modes such as MR and HR as discussed^40,43^. As in the previous cases, the LSPR peaks were gradually attenuated and broadened along with the sublimation of Ag atoms, indicating the dampening of plasmonic effect with the lower percentage of Ag. Furthermore, these LSPR peaks were broader and red shifted as compared to the previous sets. The corresponding e-field distribution, e-field vector and simulated extinction of AgAuPt NP are presented in Fig. 8(d)–(f) and Fig. S16. Similar to the previous case, the e-field intensity was generally contributed by the MR in the visible region.

With the complete desorption of Ag atoms and formation of AuPt isolated NPs, the LSPR peaks generally became much broader as shown in Fig. 8(a-2). In this case, the LSPR properties can be mainly contributed by the Au and Pt, which resulted in the broader but stronger absorption band in the VIS region than Pt NPs. The broadening effect of the VIS peak can be observed in Fig. 8(a-3). Furthermore, the e-field confinement can be seen on the AuPt NP surface in Fig. 8(g)–(i) due to the LSPR effect. It was also found that the e-field intensity of AuPt NP was reduced from the AgAuPt NP likely due to the size reduction and Ag sublimation. Similarly, the DR resonance was found to be much pronounced with the relatively smaller size of AuPt NPs. From the reflectance spectra in Fig. 8(b), it clearly showed the formation of a narrow dip in the UV region at ~ 306 nm and a wide dip in the VIS region ~ 460 nm. The reflectance dips correspond to the extinction peaks, which denotes the photon absorption due to the LSPR of AgAuPt and AuPt NPs. The reflectance dip intensity was gradually dampened with the growth of isolated nanoclusters along with the Ag sublimation^36^. Similarly, the absorption dips were further reduced with the AuPt NPs at high temperature. In terms of transmittance spectra in Fig. 8(c), it generally showed the shoulder in the UV and VIS regions for both the AgAuPt and AuPt NPs. The quadrupolar resonance (QR) can be much significant for the large size NPs, which could result in the enhanced forward scattering in the resonance wavelength, Thus, the transmittance spectra may not exhibit the absorption dips^38^.

## Conclusions

In summary, various mono-, bi- and tri-metallic nanoparticles comprising Ag, Au and Pt have been demonstrated via the solid-state dewetting of the sputtered Ag/Pt bi-layers and Ag/Au/Pt tri-layers on sapphire (0001) substrates. The systematic annealing of various multilayer films induced the solid-state dewetting and formation of well-developed mono- and multi-metallic alloy NPs based on the surface diffusion, intermixing, sublimation and energy minimization mechanism. Depending upon the temperature, the nucleation of voids, growth, nanocluster evolution and isolated NPs formation were commonly observed with the relatively thicker Ag/Au/Pt tri-layer and Ag/Pt bi-layer films. The thinner Ag/Au/Pt tri-layer showed the evolution of much uniform, dense and smaller NPs. In addition, the growth of NPs was affected simultaneously by the Ag sublimation, which tend to decrease the overall size and remove Ag content from the alloy NPs at higher temperature. Thus, above 650 °C, the AuPt and Pt NPs were realized from the Ag/Au/Pt and Ag/Pt films. These fabricated alloy and monometallic NPs exhibited the dynamic LSPR peaks in the visible region (450–700 nm) based on the surface morphology and elemental composition. For instance, the larger AuPt NPs had much broader and stronger LSPR bands whereas the small uniform AuPt NPs exhibited narrower LSPR bands. Furthermore, the LSPR band showed blue shift with the size decrement of NPs and dampening of intensity with the sublimation of Ag. Compared with the previous result of the pure Pt NPs, the LSPR response with the more isolated and definite Pt and AuPt NPs were significantly improved.

## Methods

In this work, the transparent c-plane sapphire (0001) wafers of 430 µm thickness and ±0.1° off axis were used for the fabrication of various AgAuPt, AuPt and Pt nanostructures. Initially, the sapphire wafers were diced into 6 × 6 mm^2^ squares and degassed in a pulsed laser deposition (PLD) chamber at 600 °C for 30 min under 1 × 10^−4^ Torr. This ensures the removal of trapped gases, particulates, oxides and vapors from the substrates. The bare sapphire exhibited very smooth surface morphology with the step height less 1 nm as shown in Fig. S1(a). The transmittance and reflectance showed the flat response between 300 and 1100 nm as displayed in Figs S1(b),(c) respectively.

The Ag, Au and Pt films were then deposited on a clean substrate using a plasma-assisted sputtering process. In this study, two different deposition schemes were used as shown in Fig. 1(a), namely the Ag/Pt bi-layer and Ag/Au/Pt tri-layer. Prior to the annealing, the deposited samples were placed on a holder with an additional Inconel blank on the backside to ensure the uniform heat conduction throughout the sample surface. Then, the samples were transferred to the PLD chamber for the fabrication of AgAuPt, AuPt and Pt nanostructures by thermal annealing. The annealing was performed systematically between 500 and 900 °C under the chamber pressure of 1 × 10^−4^ Torr. After attaining the base pressure, the temperature was ramped at 4 °C/s to reach the target temperature and maintained constant for 120 s as shown in Fig. S1(d). To finish the growth, the heating system was turned off without breaking the vacuum condition until the system temperature was reduced to an ambient. The annealing process was monitored by the computer recipe program for consistency.

The surface morphology of the fabricated AgAuPt, AuPt and Pt nanostructures was characterized by using a non-contact mode atomic force microscope (AFM) (XE-100, Park Systems, South Korea). The same batch of tips having the height 10 mm and radius 1 nm were used to scan samples in order to minimize the tip effect. For the further analysis, the AFM images were further processed with the XEI software (Park Systems, South Korea). In addition, the large-scale surface topologies were obtained by using a scanning electron microscope (SEM, COXEM, South Korea). Elemental characterization of the AgAuPt, AuPt and Pt nanostructures was performed by using an energy dispersive x-ray spectroscope (EDS, Thermo Fisher Scientific, United States) in a spectral mode. For the optical characterization of the AgAuPt, AuPt and Pt nanostructures, the NOST system (Nostoptiks, South Korea) equipped with the sr-500 spectrograph, optical microscope and various light source was employed. Transmittance and reflectance spectra were experimentally measured whereas the extinction spectra were calculated using the relation: R % + T % + E % = 100%.

The electromagnetic field of NPs on sapphire was simulated by using a finite-difference time domain (FDTD) solutions (Lumerical, Canada). The 3D AFM surface morphology of typical NPs was imported into the object space to incorporate the real feature of NPs. The imported NPs were then placed on sapphire, which was enclosed in the meshed region. For the excitation at normal incidence, a plane-wave source of 250–1100 nm was engaged above the NPs parallel to the z-axis. The boundary conditions in x, y and z- directions were perfectly matched layer (PML). The mesh grid size varied between 0.5 and 5 nm depending upon the size of NPs with the simulation time of 1000 fs and auto shutoff level of 10^−6^. The dielectric constant of Ag from Rakic, Au from CRC and Pt and sapphire from the Palik model by fitting in the wavelength range of 250–1100 nm^44–46^. For the alloy compositions, the complex dielectric constants were averaged from the pure metals based on the atomic % ratio^47,48^.

## Supplementary information

Supplementary Information
