# Author Correction: Strongly confined localized surface plasmon resonance (LSPR) bands of Pt, AgPt, AgAuPt nanoparticles

**DOI:** 10.1038/s41598-021-88564-2

**Published:** 2021-05-10

**Authors:** Mao Sui, Sundar Kunwar, Puran Pandey, Jihoon Lee

**Affiliations:** 1 Institute of Hybrid Materials, College of Materials Science and Engineering, Qingdao University, Qingdao, 266071 People’s Republic of China; 2 Department of Electronic Engineering, College of Electronics and Information, Kwangwoon University, Nowon-gu, Seoul, 01897 South Korea

Correction to: *Scientific Reports* 10.1038/s41598-019-53292-1, Published online 12 November 2019

The original version of this Article contained errors.

The paper omitted related works, References 6–10, which are listed below:

Pandit, S., Kunwar, S., Pandey, P. & Lee, J. Improved LSPR Properties of Ag–Pt and Pt Nanoparticles: A Systematic Study on Various Configurations and Compositions of NPs via the Solid-State Dewetting of Ag–Pt Bilayers. *Metals* **9**(9), 1011 (2019)

Sui, M., Kunwar, S., Pandey, P., Pandit, S. & Lee, J. Systematic investigation on quad-metallic AgAuPdPt and tri-metallic AuPdPt NPs through the solid-state dewetting of quad-layer Ag/Au/Pd/Pt thin films on c-plane sapphire. *PLoS one* **14**(10): e0224208 (2019)

Pandey, P., Kunwar, S., & Lee, J. Solid state dewetting of Ag/Pt bilayers for the stronger localized surface plasmon resonance (LSPR) properties: The dynamic control of surface morphology and elemental composition of AgPt and Pt nanostructures by the auxiliary Ag layer*. J. Alloys Comp.* **813**(15), 152193 (2020)

Kunwar, S., Pandey, P., Pandit, S., Sui, M., & Lee, J. Improved Morphological and Localized Surface Plasmon Resonance (LSPR) Properties of Fully Alloyed Bimetallic AgPt and Monometallic Pt NPs Via the One-Step Solid-State Dewetting (SSD) of the Ag/Pt Bilayers. *Nanoscale Res. Lett.* **14**, 332 (2019)

Pandit, S., et al*.* Fabrication of Various Plasmonic Pt Nanostructures via Indium Assisted Solid-State Dewetting: From Small Nanoparticles to Widely Connected Networks. *Nanomaterials* **9**(6), 831 (2019)

As a result, References 11–53 were originally listed as References 6–48.

The text in the Introduction,

“Multi-metallic alloy NPs can provide proficient advantages over the monometallic NPs: i.e. the multi-functionality, site specific response, dynamic surface plasmon resonance and electronic heterogeneity induced by the elemental variation^6–9^. For instance, the ultra-fine PdAg alloy NPs have exhibited the excellent catalytic activity and selectivity as compared to the Pd NPs due to the bimetallic synergy between Pd and Ag such that the Pd offers the hydrogenation activity while the Ag improves the selectivity of the target^10^. Furthermore, apart from the manipulation of size, density and structure, the variation of elemental composition in the alloy NPs can offer an additional parameter to tune the physical and chemical properties of NPs^11–13^.”

Now reads,

“Multi-metallic alloy NPs can provide proficient advantages over the monometallic NPs: i.e. the multi-functionality, site specific response, dynamic surface plasmon resonance and electronic heterogeneity induced by the elemental variation^6–14^. For instance, the ultra-fine PdAg alloy NPs have exhibited the excellent catalytic activity and selectivity as compared to the Pd NPs due to the bimetallic synergy between Pd and Ag such that the Pd offers the hydrogenation activity while the Ag improves the selectivity of the target^15^. Furthermore, apart from the manipulation of size, density and structure, the variation of elemental composition in the alloy NPs can offer an additional parameter to tune the physical and chemical properties of NPs^6–10,16–18^.”

And the text,

“On the other hand, the Pt NPs have been the promising candidates for the catalytic applications due to its strong interaction with the surrounding and chemical stability but lack of the strong LSPR response such as in the Ag and Au NPs^19,20^.”

Now reads,

“On the other hand, the Pt NPs have been the promising candidates for the catalytic applications due to its strong interaction with the surrounding and chemical stability but lack of the strong LSPR response such as in the Ag and Au NPs^6–10,24,25^.”

In addition, in the Results and discussion, the text,

“When the Pt layer was deposited on the Ag layer, there forms the Ag/Pt interface and upon annealing, some of the Ag and Pt atoms may enter into the existing vacancies at the interface, giving a rise to the partially intermixed interface. By further annealing, the atomic intermixing can be more enhanced and thus can result in the formation of fully alloyed bimetallic layer with the increased global diffusivity of atoms as depicted in Fig. 1(a-1)^30^.”

Now reads,

“When the Pt layer was deposited on the Ag layer, there forms the Ag/Pt interface and upon annealing, some of the Ag and Pt atoms may enter into the existing vacancies at the interface, giving a rise to the partially intermixed interface. By further annealing, the atomic intermixing can be more enhanced and thus can result in the formation of fully alloyed bimetallic layer with the increased global diffusivity of atoms as depicted in Fig. 1(a-1)^6–10,35^.”

These changes have been implemented in the PDF and HTML versions of the Article.

