# Supplementary material for: Strongly confined localized surface plasmon resonance (LSPR) bands of Pt, AgPt, AgAuPt nanoparticles
Source: Sci Rep. 2019 Nov 12;9:16582. doi: 10.1038/s41598-019-53292-1 (PMC6851101; doi:10.1038/s41598-019-53292-1)
Supplement: Supplementary file 1 — Supplementary Information [file 41598_2019_53292_MOESM1_ESM.docx]

**Supplementary Information**

Strongly confined localized surface plasmon resonance (LSPR) bands of Pt, AgPt, AgAuPt nanoparticles

*Mao Sui^1,2^*, Sundar Kunwar^2^, Puran Pandey^2^ and Jihoon Lee^2*^*

^1^ Institute of Hybrid Materials, College of Materials Science and Engineering, Qingdao University, Qingdao 266071, P. R. China

^2^ Department of Electronic Engineering, College of Electronics and Information, Kwangwoon University, Nowon-gu Seoul 01897, South Korea.

*Correspondence e-mail: maosui001@qdu.edu.cn; jihoonlee@kw.ac.kr

**
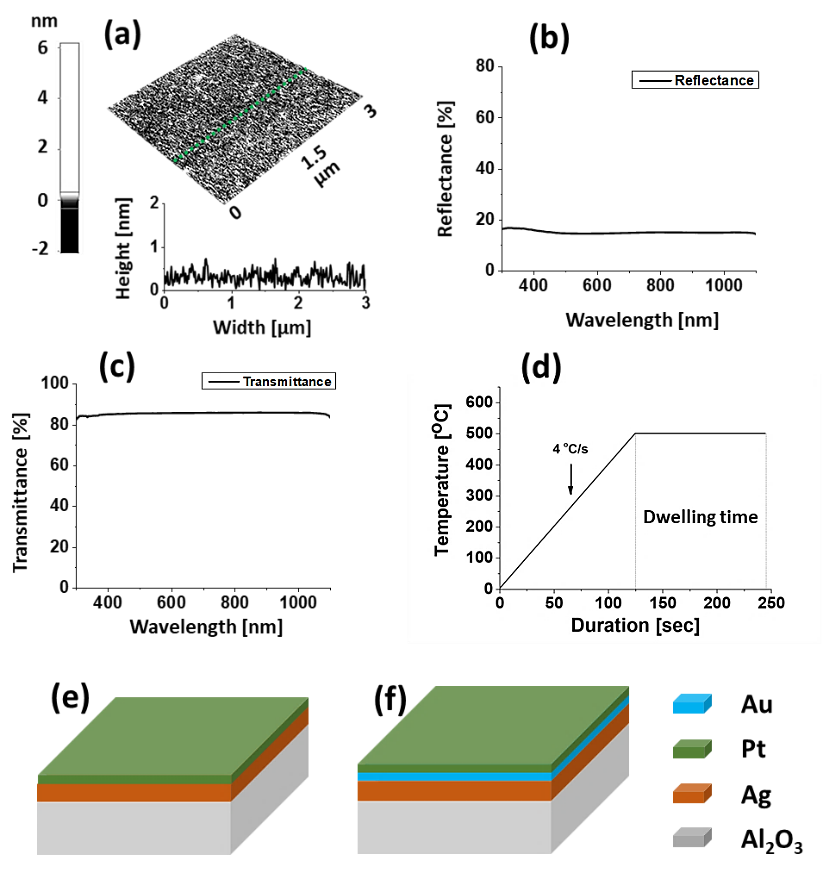
**

**Figure S1:** (a) AFM surface morphology of the bare sapphire (0001) along with the cross-sectional line profiles. The cross-sectional line profile shows the average height profile of bare sapphire below 1 nm. (b) – (c) UV-VIS-NIR (300 – 1100 nm) reflectance and transmittance spectra of bare sapphire (0001). (d) Annealing temperature graph, i.e. for the sample annealed at 500 °C for 120 s. (e) – (f) Schematic of Ag/Pt bilayer and Ag/Au/Pt tri-layers. For the Ag/Pt bilayer film, 20 nm Ag was firstly deposited and then 10 nm Pt was added atop. For the Ag/Au/Pt tri-layer films, two different thickness were adapted such as 16.5, 4.5 and 4.5 nm films of Ag, Au and Pt in the first set and 8.25, 2.25 and 2.25 nm of Ag, Au and Pt in the second set. The deposition of all three metal films was performed under 1 × 10^-1^ Torr chamber pressure with the deposition rate of 1 nm/s and ionization current of 3 mA.


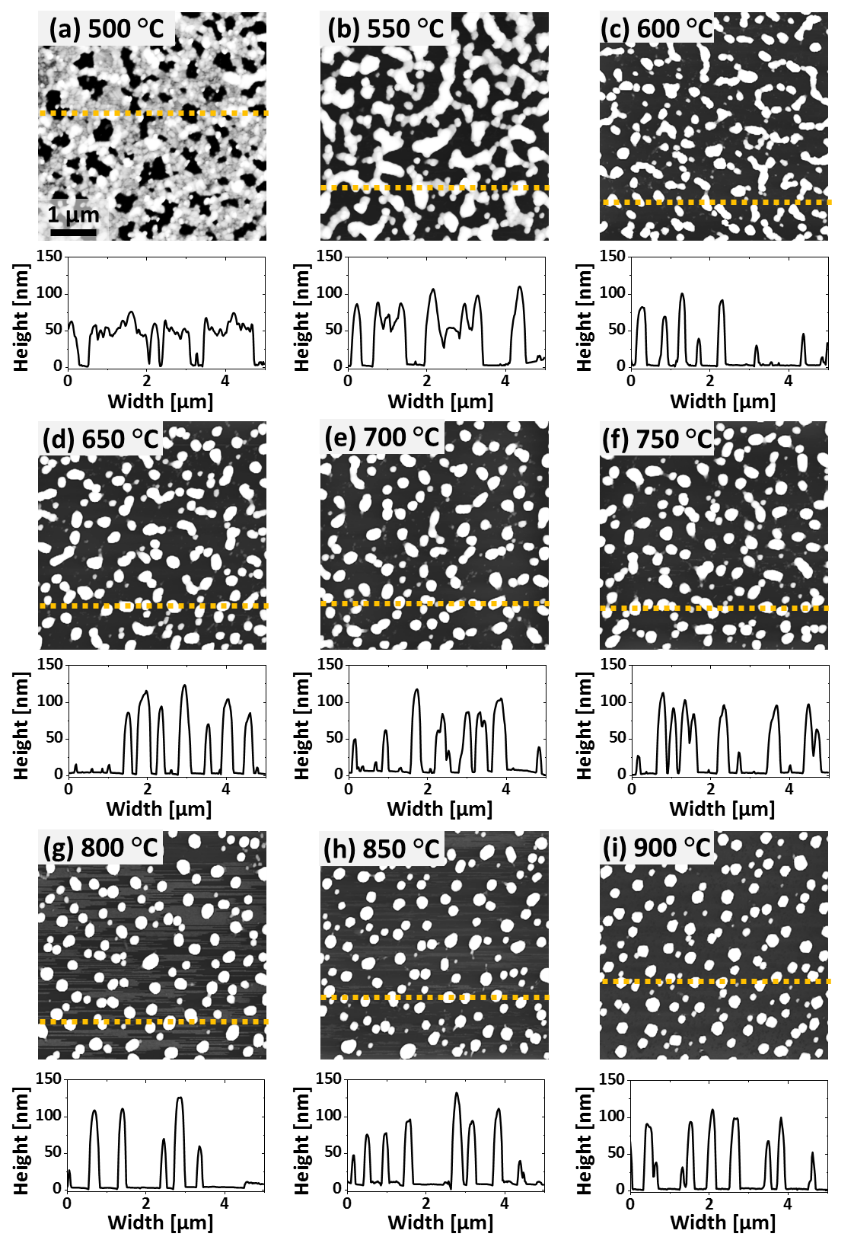


**Figure S2:** Evolution of AgPt and Pt NPs from the Ag_20nm_/Pt_10nm_ bilayers by annealing between 500 and 900 °C for 120 s. (a) – (i) AFM top-views (5 × 5 µm^2^) the NPs with corresponding line profiles.


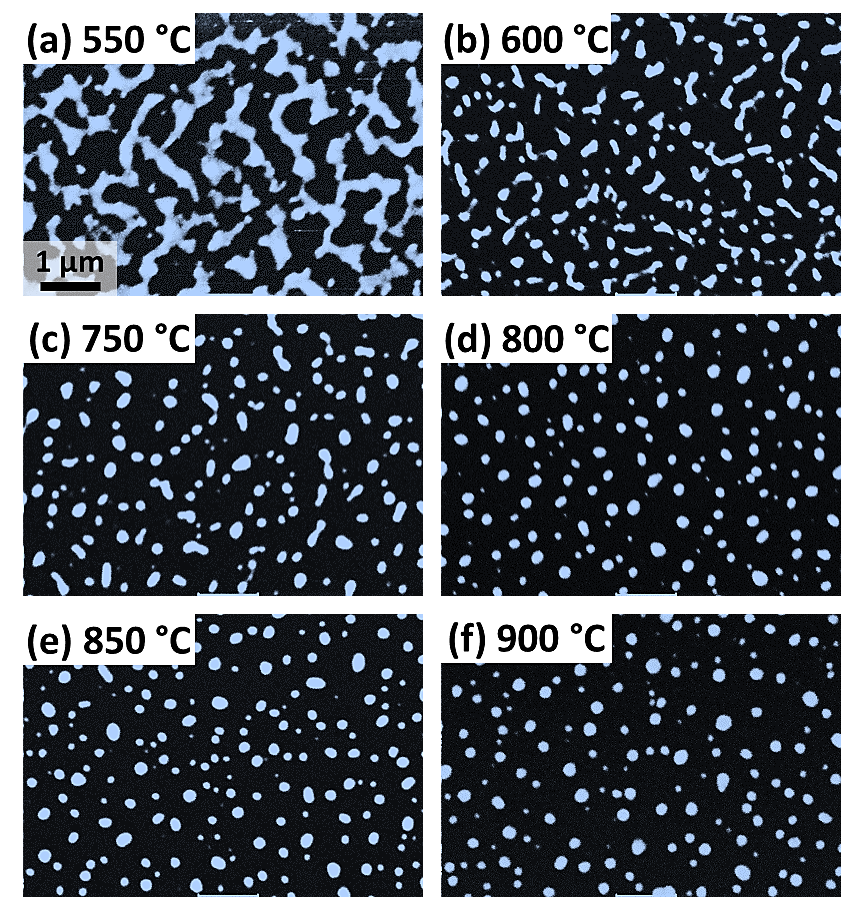


**Figure S3:** SEM images of the AgPt and Pt NPs fabricated with the Ag_20nm_/Pt_10nm_ bilayers between 550 and 900 °C for 120 s.

**
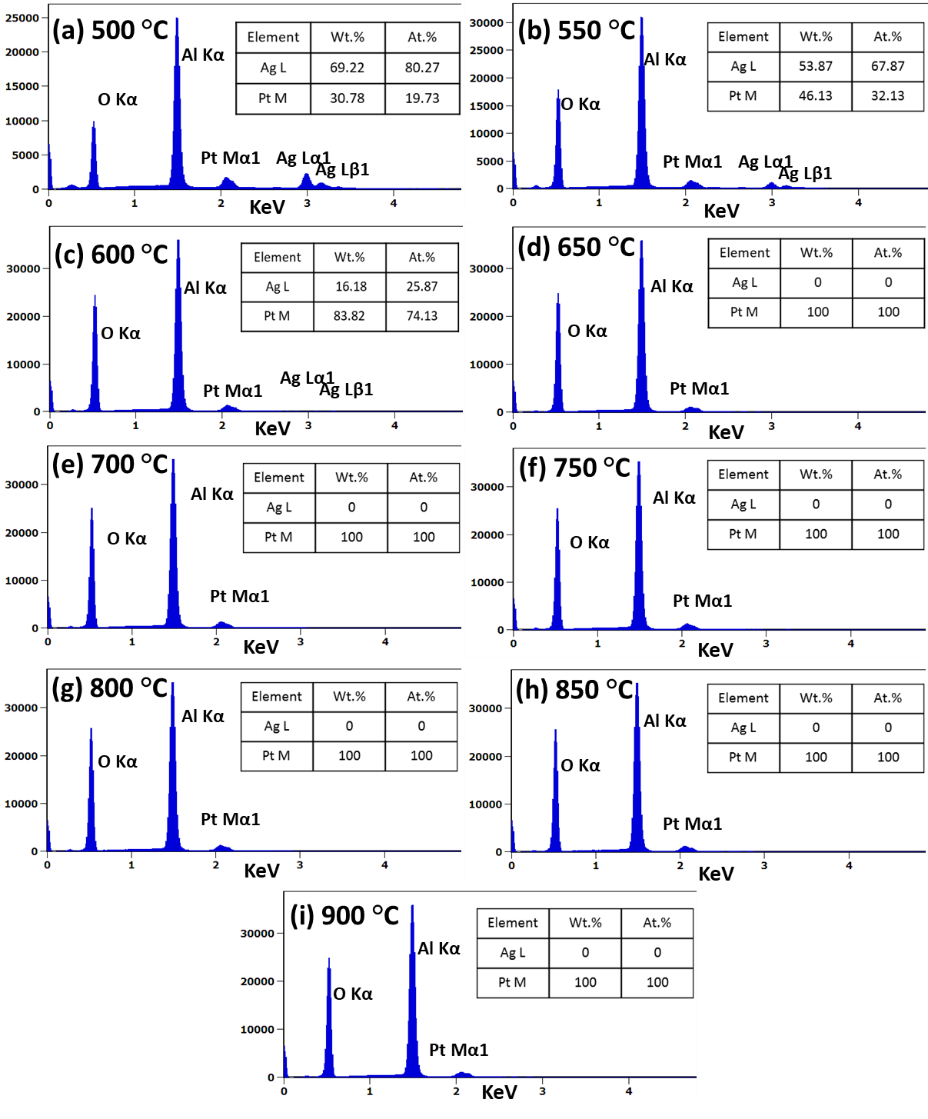
**

**Figure S4:** EDS spectra of the AgPt and Pt NPs on sapphire fabricated between 500 and 900 °C with the Ag_20nm_/Pt_10nm_ bilayers. Insets show the wt. % and at. % of Ag and Pt at different temperature.


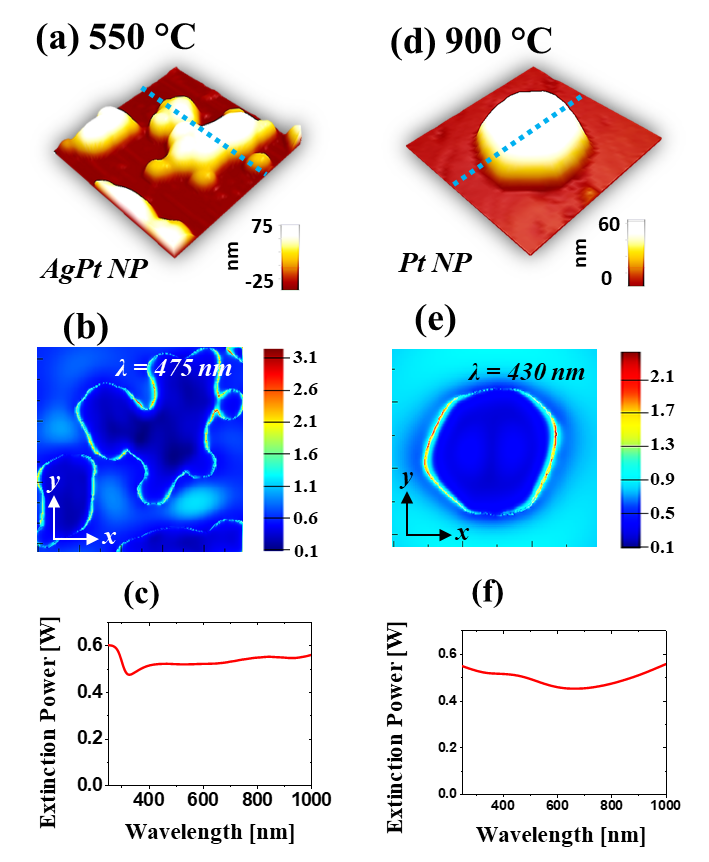


**Figure S5:** Finite difference time domain (FDTD) simulation on the typical AgPt and Pt NPs fabricated with the Ag_20nm_/Pt_10nm_ bilayers at 550 and 900 ºC. (a) and (d) AFM images of typical AgPt and Pt NPs. (b) and (e) E-field distribution at the resonance wavelength. (c) and (f) Simulated extinction power spectra.


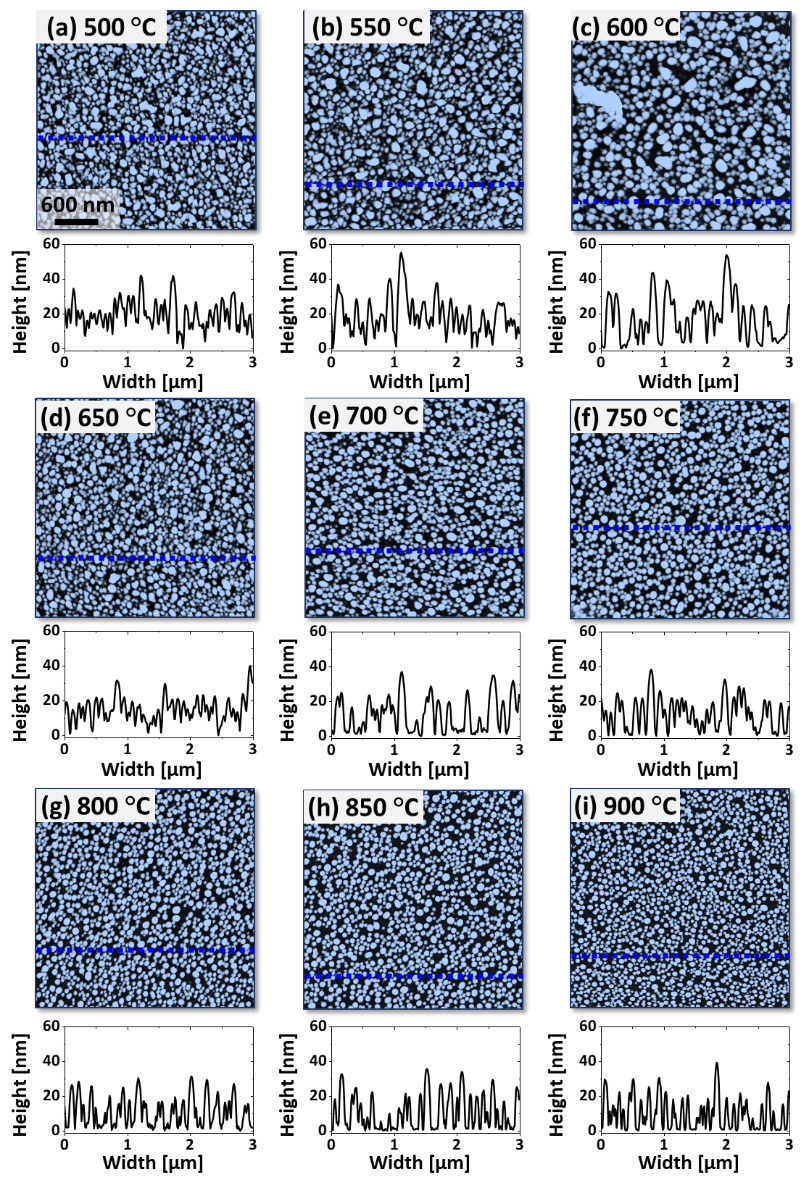


**Figure S6:** Evolution of small AgAuPt and AuPt alloy NPs by the dewetting of Ag_8.25nm_/Au_2.25nm_/Pt_2.25nm_ multilayers between 500 and 900 °C for 120 s. (a) – (i) AFM top-views (3 × 3 µm^2^) of the alloy NPs and corresponding line profiles.

**
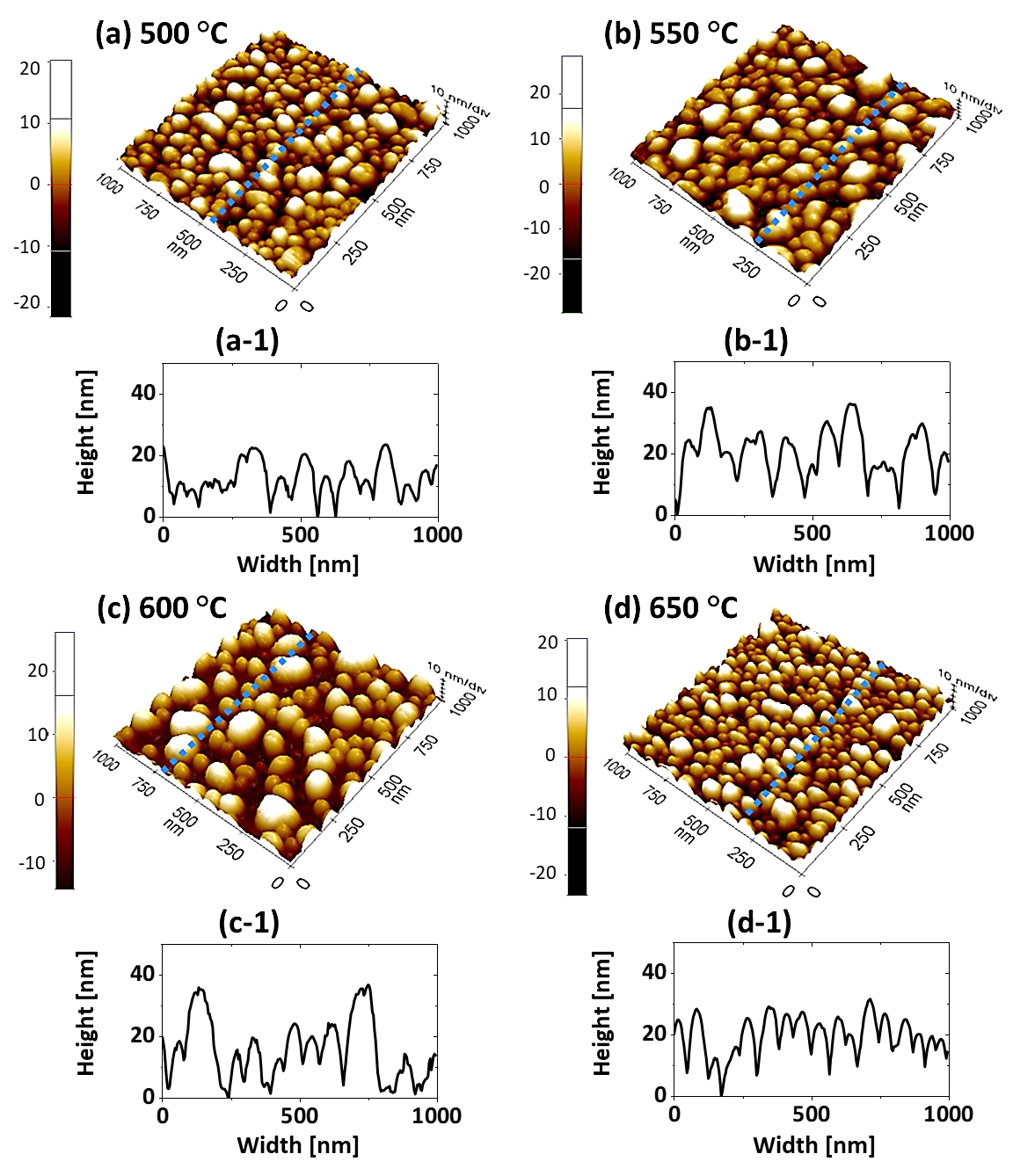
**

**Figure S7:** (a) – (c) AFM side-views (1 × 1 µm^2^) of the AgAuPt and AuPt alloy NPs fabricated with the Ag_8.25nm_/Au_2.25nm_/Pt_2.25nm_ tri-layers between 500 and 650 °C for 120 s. (a-1) – (d-1) Corresponding line profiles of the alloy NPs.

**
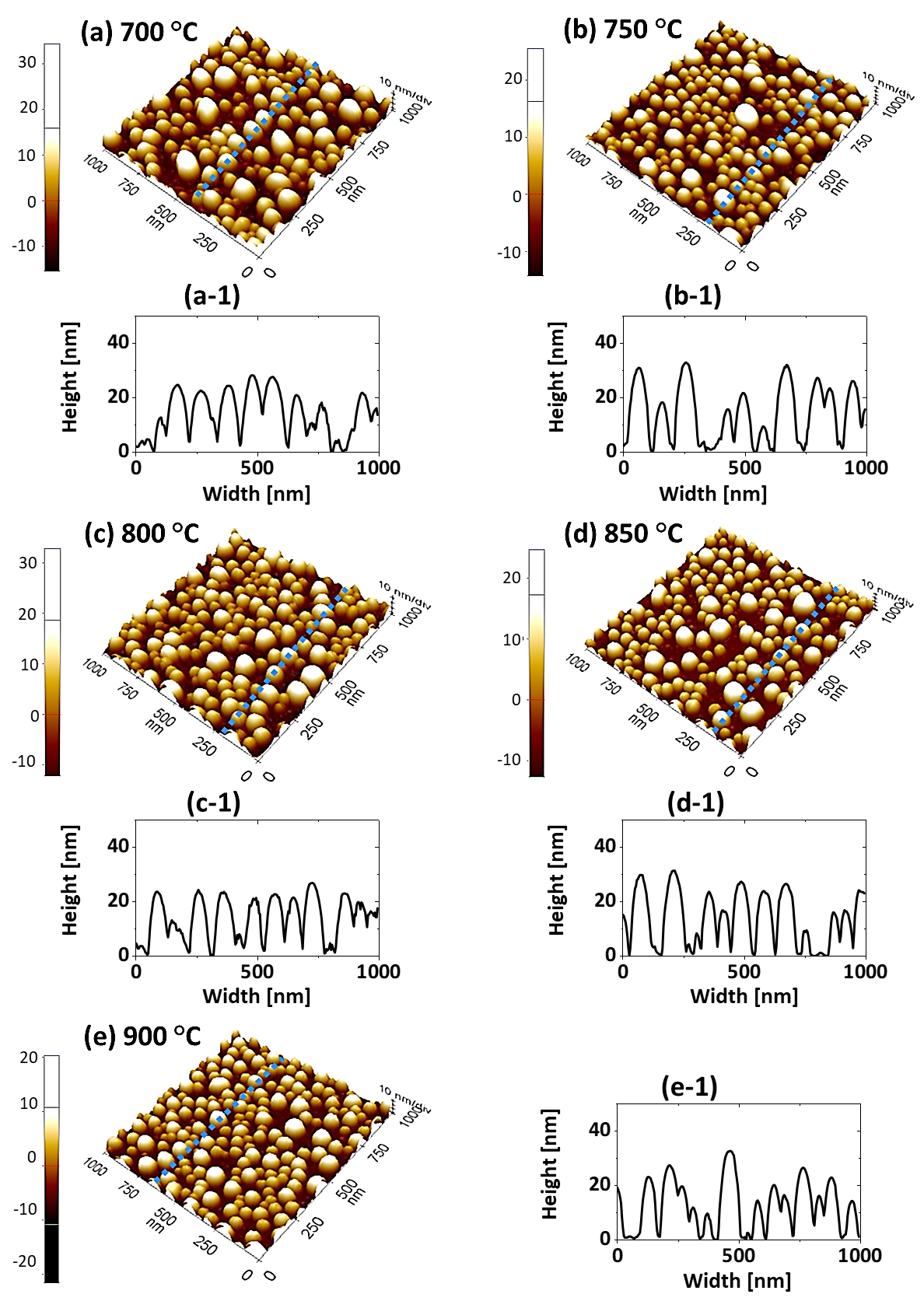
**

**Figure S8:** (a) – (c) AFM side views (1 × 1 µm^2^) of the AuPt alloy NPs fabricated with the Ag_8.25nm_/Au_2.25nm_/Pt_2.25nm_ tri-layers between 700 and 900 °C for 120 s. (a-1) – (e-1) Cross-sectional line profiles of the AuPt alloy NPs.


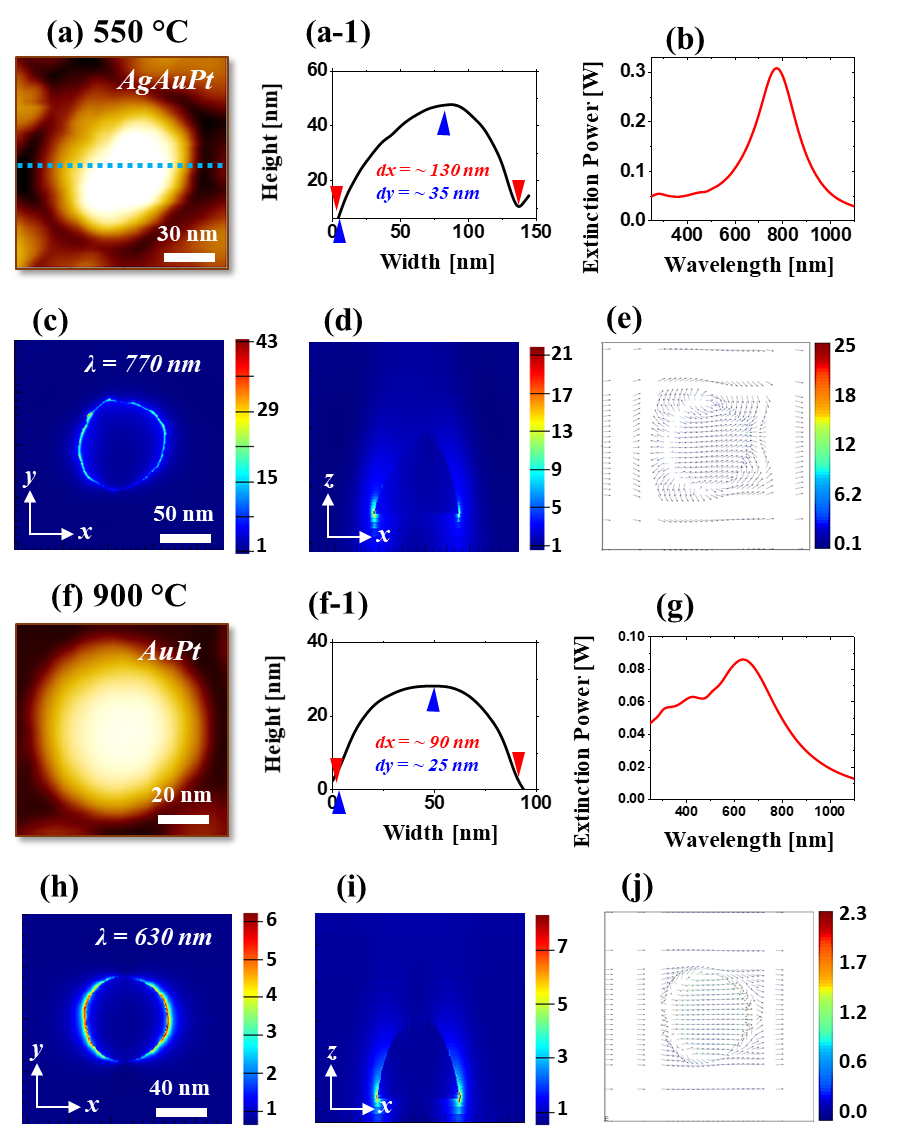


**Figure S9:** FDTD simulation of the typical AgAuPt alloy and AuPt NP on sapphire fabricated with the Ag_8.25nm_/Au_2.25nm_/Pt_2.25nm_ tri-layers at 550 and 900 °C. (a) and (f) AFM of the alloy NPs. (a-1) and (f-1) Cross-sectional line profiles. (b) – (g) Simulated extinction power spectra. (c) and (h) E-field distribution in xy-plane. (d) and (i) E-field distribution in xz-plane. (e) and (j) E-filed vector plots.

**
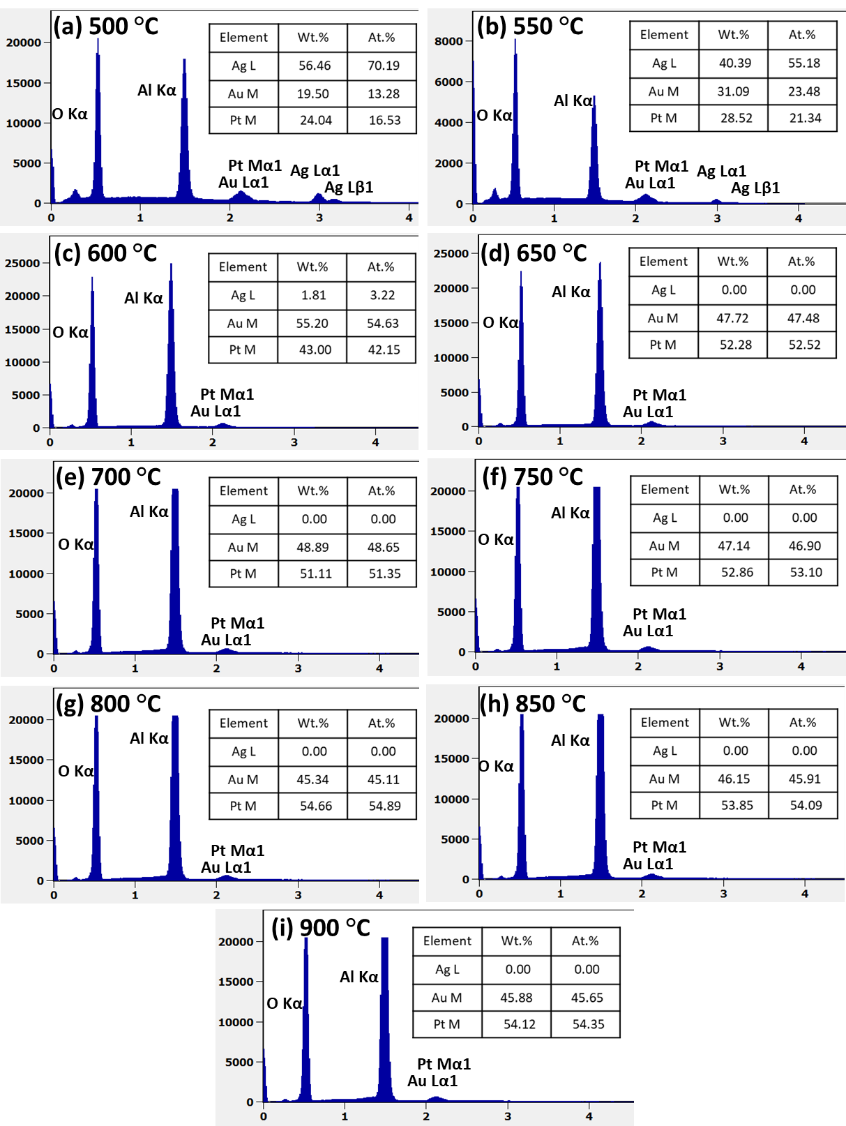
**

**Figure S10:** EDS spectra of the AgAuPt and AuPt alloy NPs on sapphire fabricated between 500 and 900 °C with the Ag_8.25nm_/Au_2.25nm_/Pt_2.25nm_ tri-layer films.


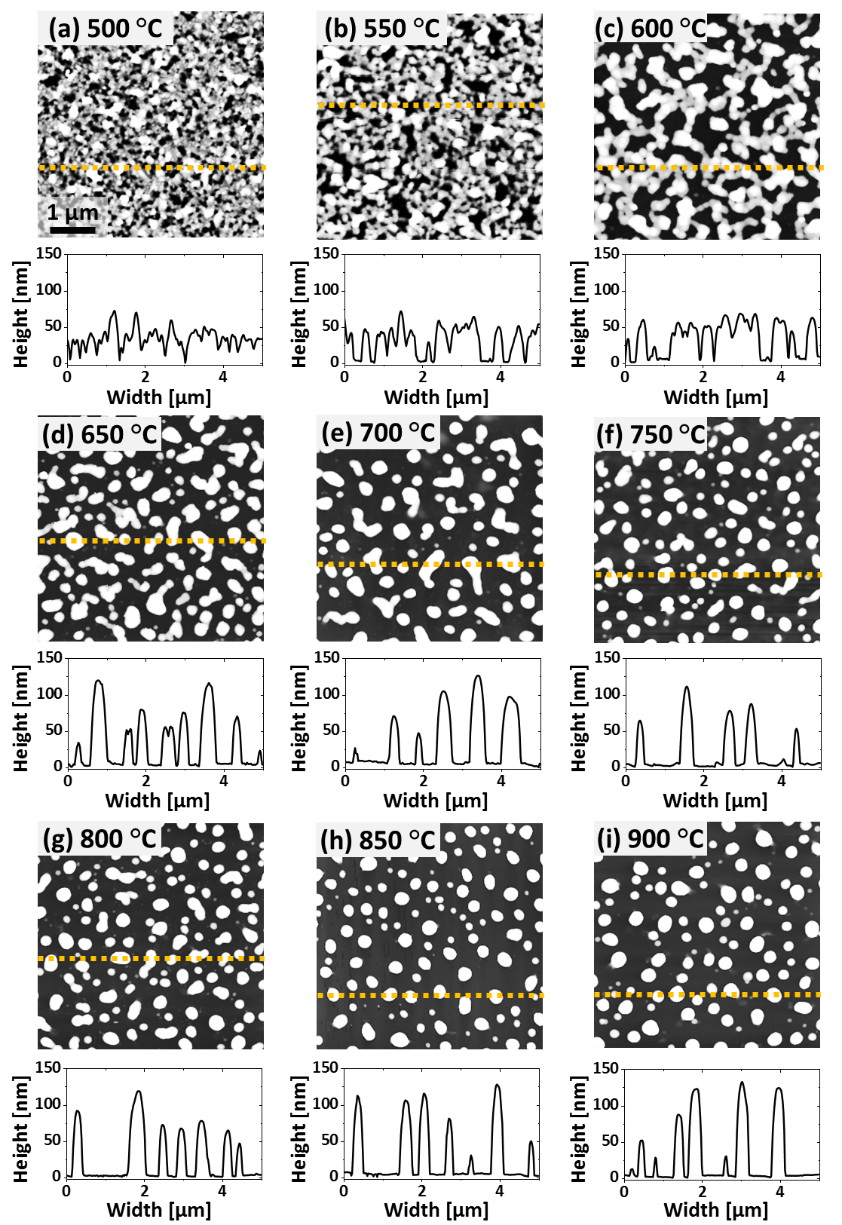


**Figure S11:** Evolution of AgAuPt alloy nanoclusters and AuPt alloy NPs by the dewetting of Ag_16.5nm_/Au_4.5nm_/Pt_4.5nm_ tri-layers between 500 and 900 °C for 120 s. (a) – (i) AFM top-views of the alloy NPs (5 × 5 µm^2^) and corresponding line profiles.


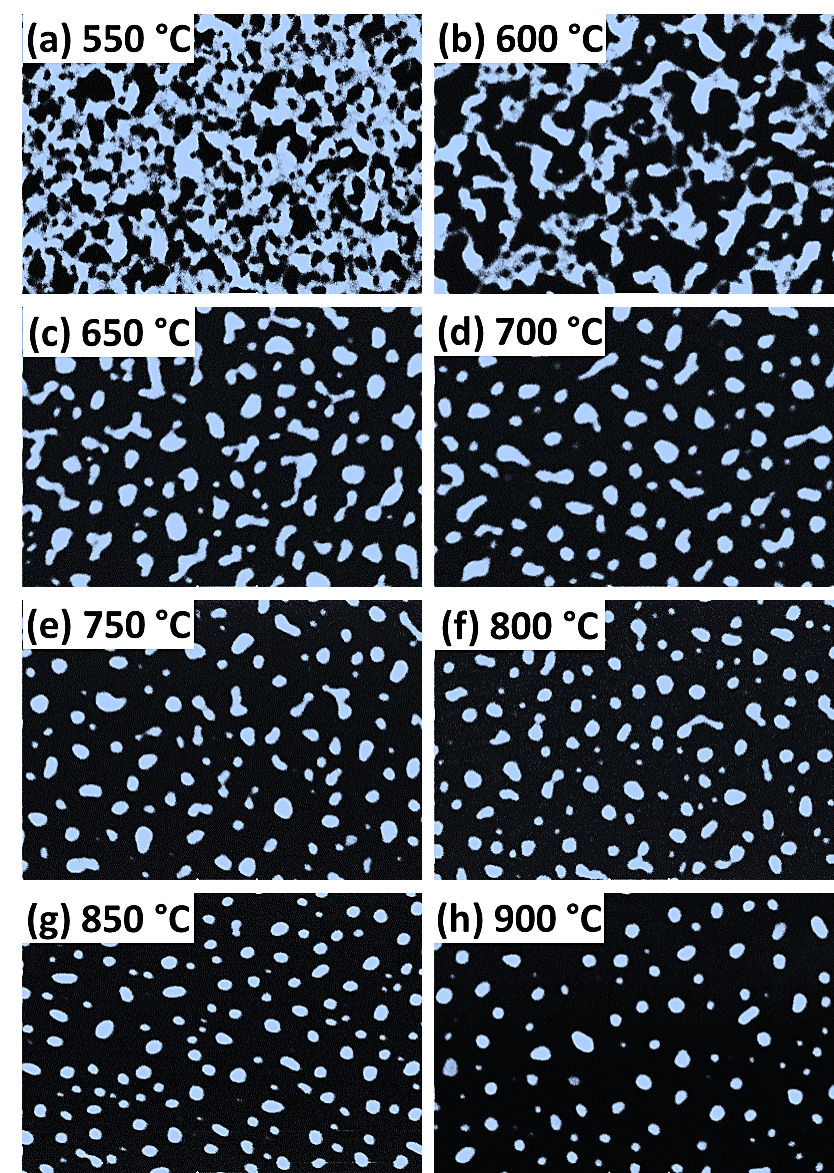


**Figure S12:** SEM images of AgAuPt alloy nanoclusters and AuPt NPs fabricated with the Ag_16.5nm_/Au_4.5nm_/Pt_4.5nm_ tri-layers by annealing between 500 and 900 °C for 120 s.


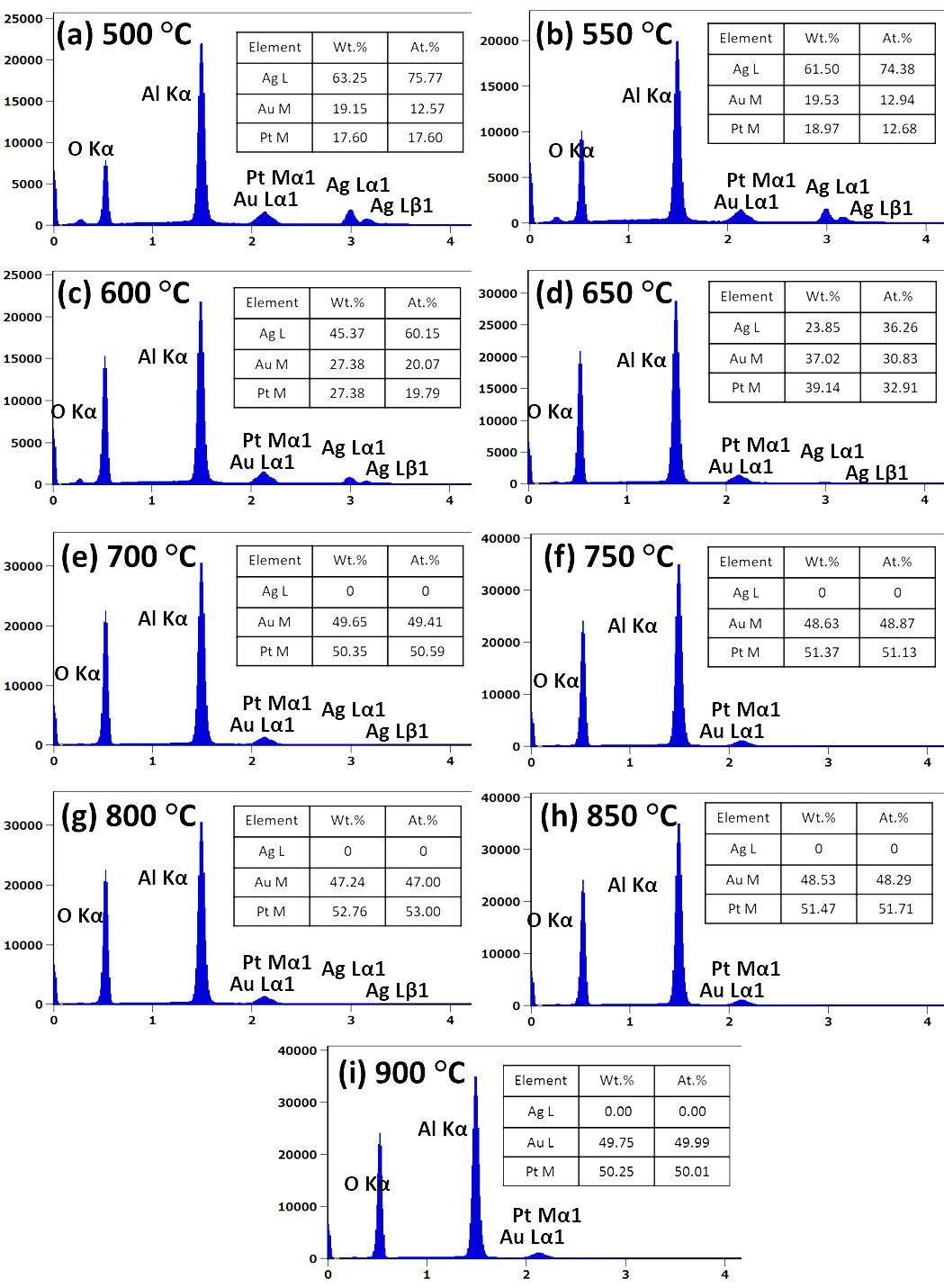


**Figure S13:** EDS spectra of the AgAuPt nanoclusters and AuPt NPs on sapphire fabricated between 500 and 900 °C with Ag_16.5nm_/Au_4.5nm_/Pt_4.5nm_ multilayers. Insets show the wt. % and at. % of Ag, Au and Pt at different temperature.


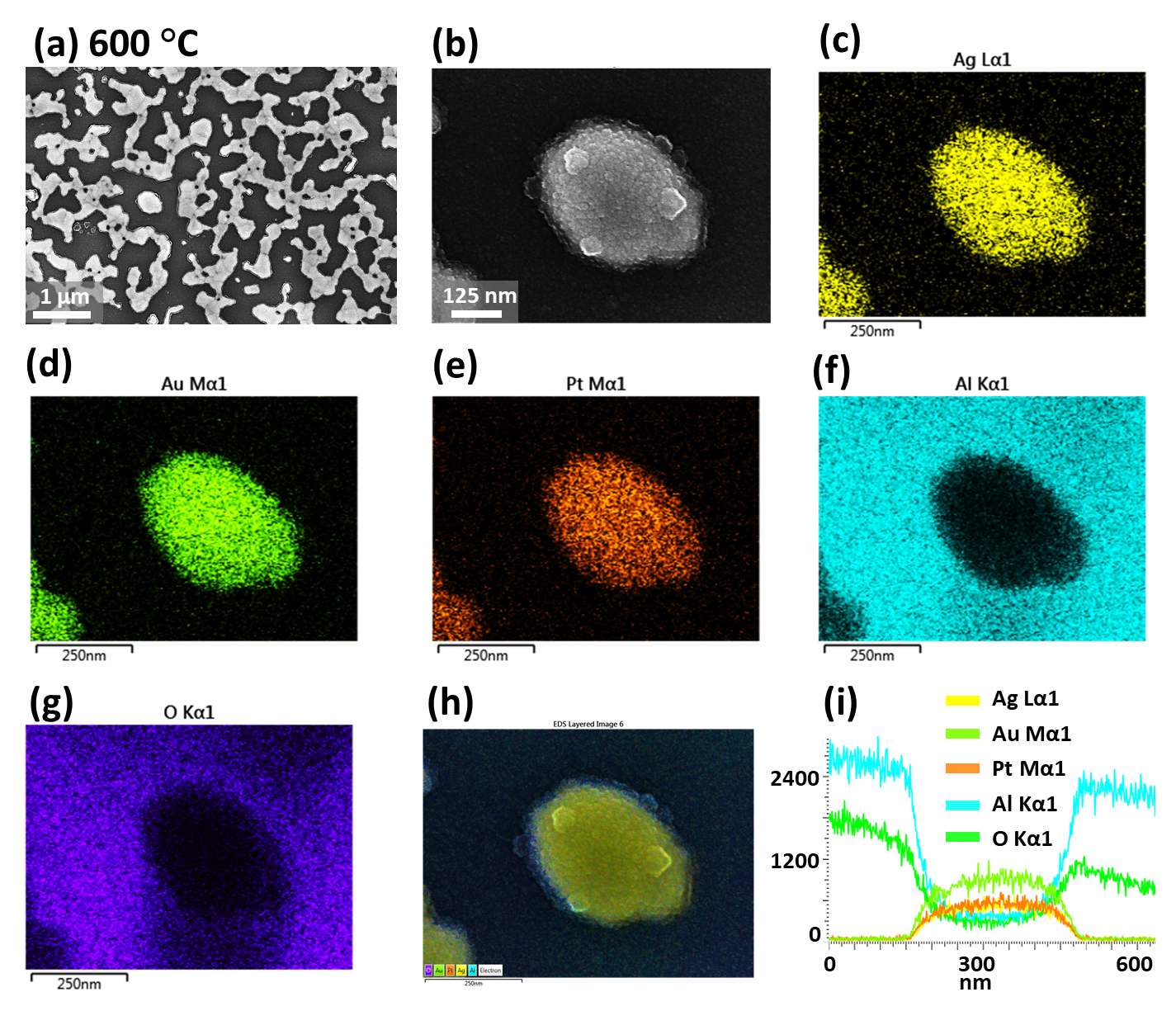


**Figure S14:** SEM images and EDS maps of multimetallic alloy nanoparticles fabricated by the annealing of Ag_16.5nm_/Au_4.5nm_/Pt_4.5nm_ multilayers at 600 ºC. (a) – (b) SEM images of the AgAuPt alloy NPs on sapphire. (c) – (g) Elemental maps of Ag, Au, Pt, Al and O. (h) Overlapped mapping of elements. (i) EDS line profiles across the NP in (b).

**
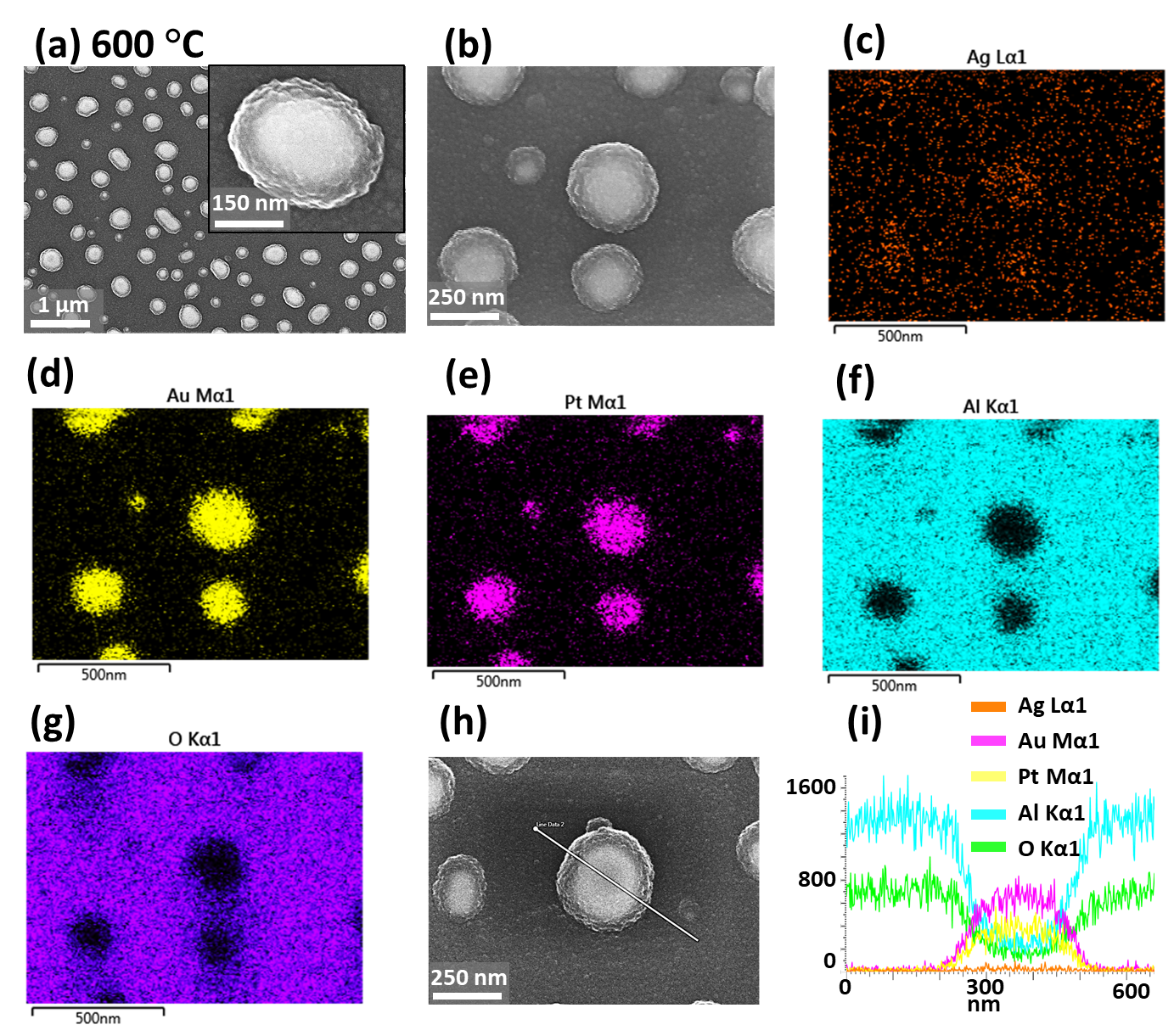
**

**Figure S15:** SEM and EDS characterization of the AuPt alloy NPs fabricated with the Ag_16.5nm_/Au_4.5nm_/Pt_4.5nm_ multilayers at 900 ºC. (a) – (b) SEM images of the AuPt alloy NPs. (c) – (g) Elemental maps of Ag, Au, Pt, Al and O. (h) – (i) SEM image and EDS line profile across the NPs. The elemental maps and line profiles clearly show the absence of Ag component while the Au and Pt are uniformly distributed in the NPs.


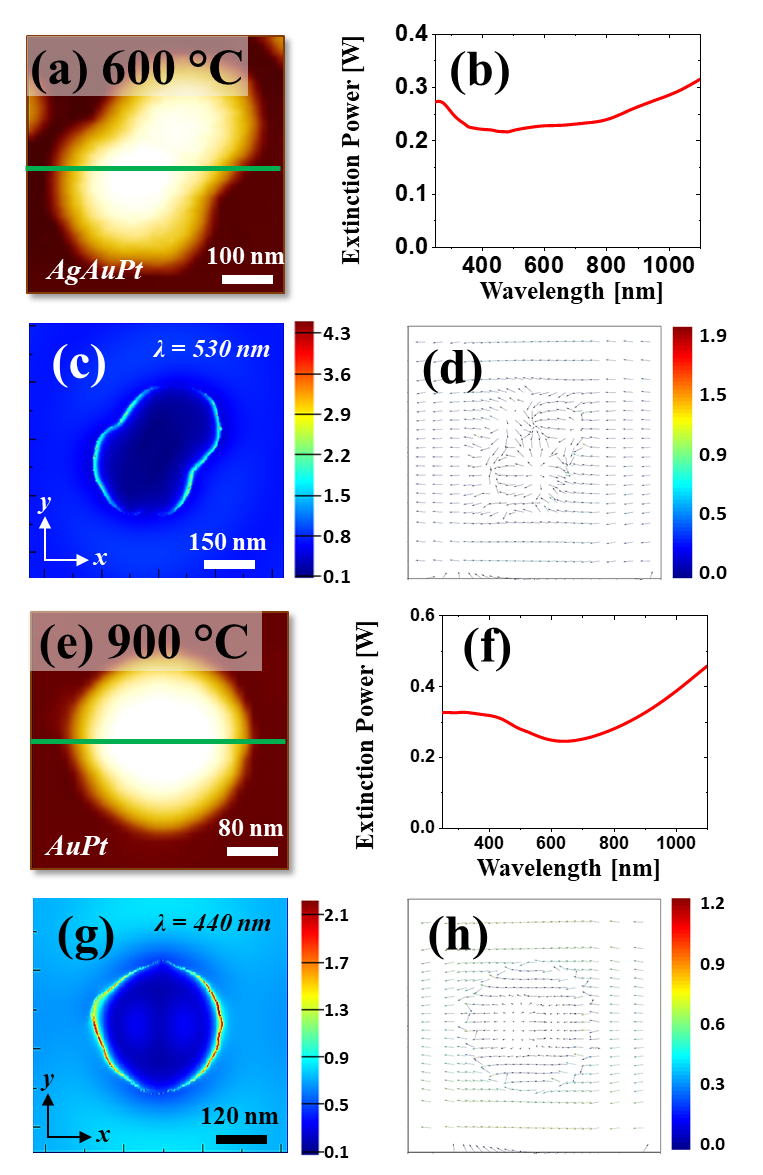


**Figure S16:** FDTD simulation the large size AgAuPt and AuPt alloy NP on sapphire fabricated with the Ag_16.5nm_/Au_4.5nm_/Pt_4.5nm_ multilayers at 600 and 900 ºC. (a) and (e) AFM top-view of the alloy NPs used for the simulation. (b) and (f) Simulated extinction power spectra. (c) and (g) E-field profile in xy-plane. (d) and (h) E-field vector plots.

**Table S1:** Summary of the Rq and SAR of the NPs fabricated with various Ag, Au, Pt multilayers, i.e. Ag_20nm_/Pt_10nm_ (type I), Ag_16.5nm_/Au_4.5nm_/Pt_4.5nm_ (type II) and Ag_8.25nm_/Au_2.25nm_/Pt_2.25nm_ (type III).

| **Temperature [°C]** | **Type I** | | **Type II** | | **Type III** | |
| --- | --- | --- | --- | --- | --- | --- |
|  | **Rq [nm]** | **SAR [%]** | **Rq [nm]** | **SAR [%]** | **Rq [nm]** | **SAR [%]** |
| **500** | 21.26 | 9.71 | 10.90 | 5.93 | 5.52 | 10.17 |
| **550** | 33.37 | 15.48 | 19.10 | 7.70 | 8.56 | 15.54 |
| **600** | 26.56 | 16.11 | 27.90 | 11.81 | 7.15 | 13.89 |
| **650** | 31.18 | 17.15 | 28.85 | 13.45 | 9.32 | 18.30 |
| **700** | 28.03 | 14.14 | 27.28 | 12.09 | 8.26 | 14.23 |
| **750** | 28.19 | 17.01 | 24.32 | 7.91 | 8.13 | 16.61 |
| **800** | 29.77 | 18.24 | 26.81 | 10.81 | 8.33 | 18.42 |
| **850** | 26.80 | 14.48 | 28.75 | 13.12 | 9.54 | 25.05 |
| **900** | 26.40 | 13.45 | 31.02 | 13.97 | 8.80 | 24.68 |

**Table S2:** Summary of average height (AH) and diameter (AD) of the NPs fabricated with the Ag_20nm_/Pt_10nm_ (type I) and Ag_16.5nm_/Au_4.5nm_/Pt_4.5nm_ (type II) films.

| **Temperature [°C]** | **type I** | | **type II** | |
| --- | --- | --- | --- | --- |
|  | **AH [nm]** | **AD [nm]** | **AH [nm]** | **AD [nm]** |
| **650** | 88 | 258 | 74 | 305 |
| **700** | 83 | 255 | 77 | 297 |
| **750** | 74 | 241 | 72 | 251 |
| **800** | 78 | 224 | 75 | 247 |
| **850** | 76 | 205 | 74 | 219 |
| **900** | 77 | 203 | 76 | 234 |
